# Causality investigation among gut microbiota, immune cells, and prostate diseases: a Mendelian randomization study

**DOI:** 10.3389/fmicb.2024.1445304

**Published:** 2024-09-11

**Authors:** Shao-Yu Yue, Wei-Yi Li, Shun Xu, Xiao-Xin Bai, Wen-Long Xu, Xu Wang, He-Kang Ding, Jia Chen, He-Xi Du, Ling-Fan Xu, Di Niu, Chao-Zhao Liang

**Affiliations:** ^1^Department of Urology, The First Affiliated Hospital of Anhui Medical University, Anhui Medical University, Hefei, China; ^2^Institute of Urology, Anhui Medical University, Hefei, China; ^3^Anhui Province Key Laboratory of Urological and Andrological Diseases Research and Medical Transformation, Anhui Medical University, Anhui Medical University, Hefei, China; ^4^Department of Infectious Disease, The Second People’s Hospital of Fuyang City, Fuyang, China

**Keywords:** prostate diseases, immune cells, mediating effect, Mendelian randomization analysis, prostate cancer, prostatitis, benign prostatic hyperplasia

## Abstract

**Background:**

The gut microbiota has been demonstrated to have a significant role in the pathogenesis and progression of a variety of diseases, including prostate cancer, prostatitis, and benign prostatic hyperplasia. Potential links between prostate diseases, immune cells and the gut microbiota have not been adequately investigated.

**Methods:**

MR studies were conducted to estimate the effects of instrumental variables obtained from genome-wide association studies (GWASs) of 196 gut microbial taxa and 731 immune cells on the risk of prostate diseases. The primary method for analysing causal relationships was inverse variance-weighted (IVW) analysis, and the MR results were validated through various sensitivity analyses.

**Results:**

MR analysis revealed that 28 gut microbiome taxa and 75 immune cell types were significantly associated with prostate diseases. Furthermore, reverse MR analysis did not support a causal relationship between prostate diseases and the intestinal microbiota or immune cells. Finally, the results of the mediation analysis indicated that Secreting Treg % CD4 Treg, Activated & resting Treg % CD4 Treg, and Mo MDSC AC inhibited the role of the class Mollicutes in reducing the risk of PCa. In prostatitis, CD8+ T cells on EM CD8br hinder the increased risk associated with the genus *Eubacterium nodatum* group. Interestingly, in BPH, CD28- CD25++CD8br AC and CD16-CD56 on HLA DR+ NK promoted the role of the genus Dorea in reducing the risk of BPH.

**Conclusion:**

This study highlights the complex relationships among the gut microbiota, immune cells and prostate diseases. The involvement of the gut microbiota in regulating immune cells to impact prostate diseases could provide novel methods and concepts for its therapy and management.

## 1 Introduction

Prostate diseases encompass a range of urinary system disorders that significantly affect the overall health of males. The prostate is a vital component of the male reproductive system and plays a pivotal role in diverse physiological processes. Prostatitis is a prevalent condition that affects the urinary system. The prevalence of symptoms related to prostatitis in males varies between 2.2 and 9.7%, with an average prevalence ranging from 8 to 8.2% (Krieger et al., 2008). Benign prostatic hyperplasia (BPH) is a benign condition marked by the proliferation of prostate tissue. The prostate grows as people age, which can compress the urethra and cause urinary symptoms like frequency, urgency, and decreased flow. The prevalence of BPH has increased 17% over the course of the past 80 decades (Launer et al., 2021), and this increase is expected to persist due to population aging. Prostate cancer (PCa), the second most prevalent cancer and the fifth leading cause of cancer-related mortality in men (Bray et al., 2018; Sung et al., 2021), varies with region and ethnicity (Siegel et al., 2020; Zhu et al., 2020; Abraham-Miranda et al., 2021; Graham-Steed et al., 2013). PCa is one of the most common malignancies in males and usually grows slowly but can advance quickly in some circumstances (Czyż et al., 2012; Folcher et al., 2023). Hence, timely identification and intervention might mitigate the consequences of prostate disease on patients’ health.

The gut microbiota is essential for the immune response, metabolism, development, and other physiological functions. The relationship between the gut microbiota and prostate diseases has been a subject of increasing interest in research, with evidence suggesting that gut microbiota dysbiosis may contribute to immune system dysregulation and exacerbate inflammation in prostatitis patients (Wastyk et al., 2021). Studies have shown notable disparities in the gut microbiota composition between chronic prostatitis and controls (Shoskes et al., 2016; Wang et al., 2023). Moreover, the gut microbiota can generate metabolites that may affect prostatitis (Du et al., 2020; Qiao et al., 2023; Chen et al., 2020). Specific microbes can produce hormone-like compounds, including short-chain fatty acids, that can potentially disrupt the typical function of the prostate (Du et al., 2022). Despite some initial evidence and theoretical justification linking the gut microbiota to prostate diseases, more complete study is needed to determine the processes and causal links (Liu et al., 2021). Alterations in the composition of the gut microbiota and the abundance of various gut microbes have been documented in individuals diagnosed with BPH (Takezawa et al., 2021; Li et al., 2022). Recent findings have revealed a potential link between periodontitis and the development of BPH through the influence of the gut microbiota and its byproducts (Guo et al., 2023). Additionally, the use of sodium butyrate may relieve BPH symptoms (Dong et al., 2022), and an imbalance in the Firmicutes/Bacteroidetes ratio has been linked to prostate enlargement (Takezawa et al., 2021). These findings imply that alterations in the gut microbiota have significant implications for the diagnosis, treatment, and early prevention of BPH. Emerging evidence suggests a possible link between the gut microbiota and cancer susceptibility (Matsushita et al., 2021; Matsushita et al., 2023), with gut dysbiosis exacerbating cancer progression (Zhong et al., 2022). The modulation of the gut microbiota may contribute to PCa development by influencing the insulin-like growth factor 1 (IGF1) signaling pathway through short-chain fatty acids, as well as by inducing autophagy in cancer cells and promoting the polarization of M2 cells (Matsushita et al., 2021; Liu et al., 2023). Hence, it is imperative to adopt a novel methodology for investigating the causal relationships between the gut microbiota and prostate diseases.

Mendelian randomization (MR) is used to combine summary data from genome-wide association studies (GWASs) to determine causal associations between exposures and outcomes (Davies et al., 2018). GWASs have played a pivotal role in the identification of genetic variants linked to diseases, particularly single nucleotide polymorphisms (SNPs), contributing to our comprehension of the genetic underpinnings of various complex traits in human diseases (Smith and Ebrahim, 2003). MR enables the estimation of causal effects between “exposure factors” and “outcomes” (Pierce and Burgess, 2013). The utilization of large-scale summary statistics in investigating the associations between the gut microbiota or immune cells and prostate diseases has significantly improved two-sample MR analysis.

The aim of this study was to investigate the possible causal connection between the gut microbiota, immune cells, and prostate diseases. Employing genome-wide association datasets for MR analysis will enhance the understanding of the intricate origins of prostate diseases and identify possible treatment targets. The involvement of gut microbes and immune cells may provide novel insights and methods for preventing, treating, and managing prostate diseases.

## 2 Materials and methods

### 2.1 Study design

**Figure 1:**
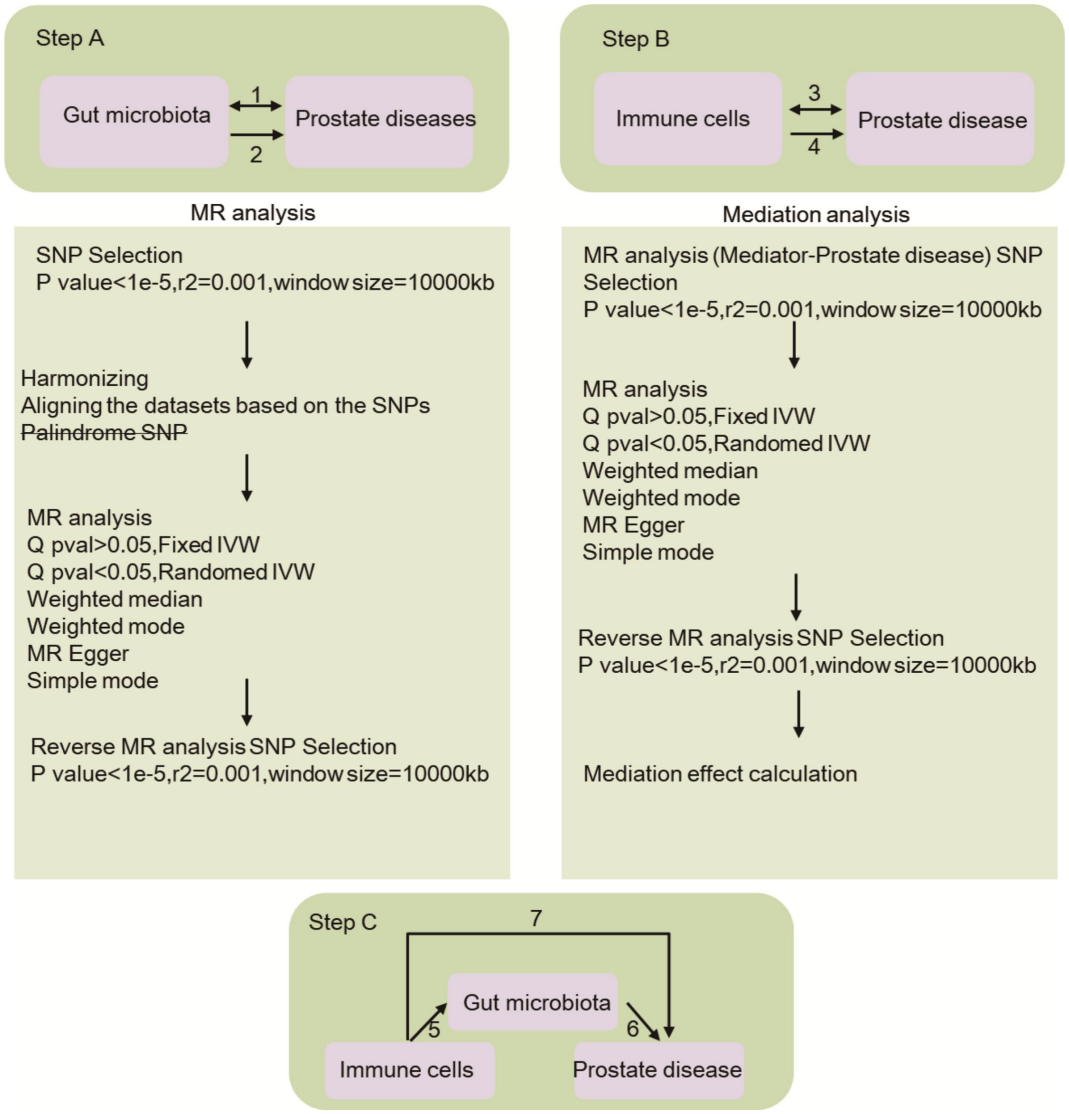
This research provides an overview of the causal impact of gut microbiota and immune cells on prostate diseases, with Step A2 illustrating the former and Step B4 illustrating the latter. Additionally, the bidirectional causal relationship between gut microbiota and prostate diseases is depicted in Step A1, while the same relationship between immune cells and prostate diseases is shown in Step B3. Step C involves the mediation analysis of immune cells in the pathway linking gut microbiota to prostate diseases. Specifically, Path 7 examines the overarching impact of gut microbiota on prostate diseases, Path 6 explores the causal relationship between immune cells and prostate diseases, and Path 5 investigates the causal effect of gut microbiota on immune cells.

This study encompasses three primary components, as depicted in Figure 1: the investigation of the causal effects of 196 gut microbial taxa on three categories of prostate diseases (Step 1A); the analysis of the causal effects of 731 immune cells on three types of prostate diseases (Step 2A); and the implementation of mediation analysis to examine the role of the gut microbiota in mediating the pathway from immune cells to prostate diseases (Step 3). SNPs were defined as instrumental variables (IVs) (Bowden and Holmes, 2019).

### 2.2 Data source

The GWAS summary data comprise 196 gut microbial taxa categorized into 119 genera, 32 families, 20 orders, 16 classes, and 9 phyla. The genetic information for the gut microbiome was obtained from the latest GWAS summary data, which underwent meticulous curation and analysis by the esteemed MiBioGen consortium. The present analysis employed whole-genome genotype and 16S fecal microbiome data from a cohort of 18,340 individuals across 24 distinct studies (Kurilshikov et al., 2021). Accession numbers ranging from GCST0001391 to GCST0002121 in the GWAS Catalog provide public access to immune cell data (Orrù et al., 2020). A comprehensive examination of 731 immunophenotypes was conducted in this study. The summary data for prostatitis, PCa, and BPH can be found in the GWAS Catalog under the accession numbers GCST90084104, GCST90079112, and GCST90081804, respectively. In this study, a secondary analysis of publicly available GWAS summary statistics was conducted, with ethical approval obtained for each original GWAS. Furthermore, the study did not involve the use of individual-level data, thus obviating the need for additional ethical approval from an ethics review committee.

### 2.3 Selection of IVs

Initially, SNPs demonstrating significant associations with the gut microbiota (*p* < 1 × 10^-5) were chosen for analysis. SNPs exhibiting linkage disequilibrium (LD) were omitted, with a predetermined threshold of *p* < 5 × 10^^−6^. The LD between the identified SNPs and the gut microbiota was required to meet the criteria of r^^2^ < 0.001 and a distance exceeding 10,000 kb (Myers et al., 2020). A crucial component of MR analysis involves verifying that the impact of an SNP on an individual’s exposure aligns with the effect of the SNP on the outcome for the same allele. Following this comparison, palindromic SNPs (defined as those with A/T or G/C alleles) were subsequently excluded from the analysis.

### 2.4 MR analysis

#### 2.4.1 Primary analysis

To evaluate the potential causal association between the gut microbiota and immune cells in relation to prostate diseases, we conducted independent two-sample MR analyses (steps 1A and 2A in Figure 1). The analytic methods employed were IVW, MR-Egger, weighted median, simple mode, and weighted mode, and Wald ratio tests were conducted for variables featuring only one IVW result (Pierce and Burgess, 2013). In cases where discrepancies arose between these methods, the IVW results were considered the primary outcome. IVW is a method for calculating a weighted average for random variable analysis. One uses the variance of every random variable as a weight. This approach can help to lower the mean’s fluctuation. SNPs are employed to simulate the genetic grouping process. This randomizing strategy allows one to overcome the usual linear character of genetic variation and other potential factors, producing more exact causal inference. The MR–Egger test was utilized to identify outliers and assess horizontal pleiotropy. By considering the similarity in causality, a weighted model technique may categorize SNPs into distinct subsets and utilize the subset with the greatest number of SNPs to evaluate the causal connection between exposure and result. Thus, funnel plots were employed to assess potential directional pleiotropy. To mitigate the potential inaccuracies caused by SNPs, a leave-one-out study was conducted to evaluate the impact of individual SNPs on the MR estimates. Meanwhile, we use website tools^1^ to eliminate confounding factors. All Mendelian randomization analyses were performed via the ‘Two Sample MR’ (version 0.5.6) package in R version 4.3.1, setting statistical significance at *p* < 0.05.

#### 2.4.2 Mediation analysis

By utilizing the two-sample analysis techniques outlined in steps 3 and 4 of Figure 1, we integrated gut microbiota and immune cells that were significantly correlated with prostate diseases into a mediation analysis framework. Our investigation focused on determining the causal relationship between gut microbiota and immune cells (step 3 in Figure 1, path 5). Subsequently, to evaluate the potential mediating role of immune cells in the pathway linking the gut microbiome and prostate diseases, we conducted multiple MR analyses.

#### 2.4.3 Bidirectional causality analysis

To evaluate the bidirectional causal connections among gut microbiota, immune cells, and prostate diseases, we designated prostate diseases as the “exposure” and identified gut microbiota linked to prostate diseases as the “outcome” (steps 1 and 3 in Figure 1). We specifically chose SNPs that exhibited significant associations with prostate diseases (*p* < 5 × 10^^−8^) as IVs.

### 2.5 Sensitivity analysis

Cochran’s Q test was utilized to evaluate heterogeneity for each SNP (Bowden and Holmes, 2019), and scatter plots were generated to illustrate the MR results of SNP-exposure and SNP-outcome associations. We employed the stepwise elimination method to assess the individual impact of each SNP pair on the outcomes, wherein we systematically excluded one SNP at a time and applied the IVW method to the remaining SNPs to evaluate the influence of the specific variants on the estimated values.

## 3 Results

### 3.1 IV selection

After undergoing a rigorous quality control process, the F-statistic values for gut microbiota all exceeded the threshold of >10, suggesting a reduced susceptibility to weak instrument bias. In total, a total of 412 SNPs were identified as being associated with 28 gut microbiota taxa at various taxonomic levels, with a significance level of *p* < 1 × 10^−5^ (Supplementary Table S1). Additionally, a total of 1,650 SNPs were found to be associated with 75 immune cell types at a significance level of *p* < 5 × 10^−6^ (Supplementary Table S2).

### 3.2 Prostate cancer

#### 3.2.1 Associations between the gut microbiota and PCa

**Figure 2:**
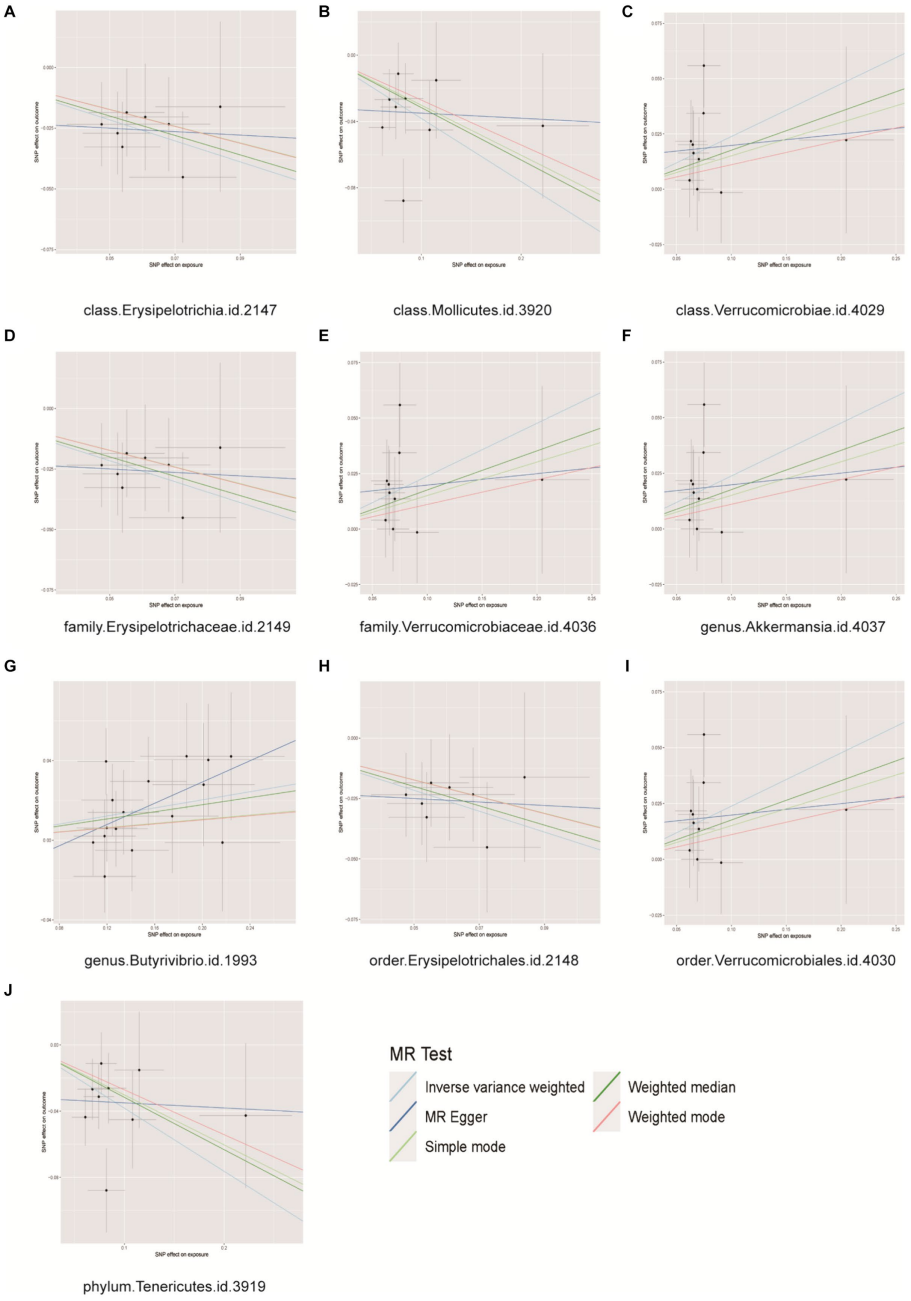
Scatter plots for causal effects of gut microbes on prostate cancer. **(A)** Class Erysipelotrichia, **(B)** class Mollicutes, **(C)** class Verrucomicrobiae, **(D)** family Erysipelotrichaceae, **(E)** family Verrucomicrobiaceae, **(F)** genus Akkermansia, **(G)** genus Buutyrivibrio, **(H)** order Erysipelotrichales, **(I)** order Verrucomicrobiales, **(J)** phylum Tenericutes.

Prostate cancer is associated with 10 distinct types of gut microbiota from three classes, two families, two genera, two orders, and one phylum. Detailed information regarding 124 SNPs for these 10 gut microbiota types can be found in Supplementary Table S3. The MR analysis (Figure 2) indicated a correlation between genetic predictions of five gut microbiota (class Verrucomicrobiae, family Verrucomicrobiaceae, order Verrucomicrobiales, genus Akkermansia, and genus Butyrivibrio) and an elevated risk of PCa. The prevalences of the class Verrucomicrobiae [odds ratio (OR) = 1.2078, 95% confidence interval (CI) = 1.0410–1.4012, *p* = 0.0127], family Verrucomicrobiaceae (OR = 1.2078, 95% CI = 1.0410–1.4012, *p* = 0.0127), order Verrucomicrobiales (OR = 1.2078, 95% CI = 1.0410–1.4012, *p* = 0.0127), genus Akkermansia (OR = 1.2076, 95% CI = 1.0409–1.4009, *p* = 0.0128), and genus Butyrivibrio (OR = 1.1068, 95% CI = 1.029–1.1904, *p* = 0.0063) were found to be significantly elevated in cases of PCa. The identification of genetic markers within five distinct intestinal microbiota groups (class Erysipelotrichia, class Mollicutes, family Erysipelotrichaceae, order Erysipelotrichales, and phylum Tenericutes) has been linked to a decreased risk of PCa. The results indicated that the presence of class Erysipelotrichia (OR = 0.7762, 95% CI = 0.6418 ~ 0.9387, *p* = 0.0090), class Mollicutes (OR = 0.7621, 95% CI = 0.6324 ~ 0.9182, *p* = 0.0042), family Erysipelotrichaceae (OR = 0.7762, 95% CI = 0.6418 ~ 0.9387, *p* = 0.0090), order Erysipelotrichales (OR = 0.7762, 95% CI = 0.6418 ~ 0.9387, *p* = 0.0090), and phylum Tenericutes (OR = 0.7621, 95% CI = 0.6324 ~ 0.9182, *p* = 0.0042) was associated with a significantly reduced risk of PCa. Importantly, our findings demonstrate heterogeneity, pleiotropy, and sensitivity, including in MR–Egger and weighted median (WM) analyses, which largely corroborated the primary results by consistently indicating the same trends (Supplementary Figures S1–S3). Conversely, reverse MR analysis did not yield any statistically significant findings, as evidenced by the results presented in Supplementary Table S4.

#### 3.2.2 Associations between immune cells and prostate cancer

**Figure 3:**
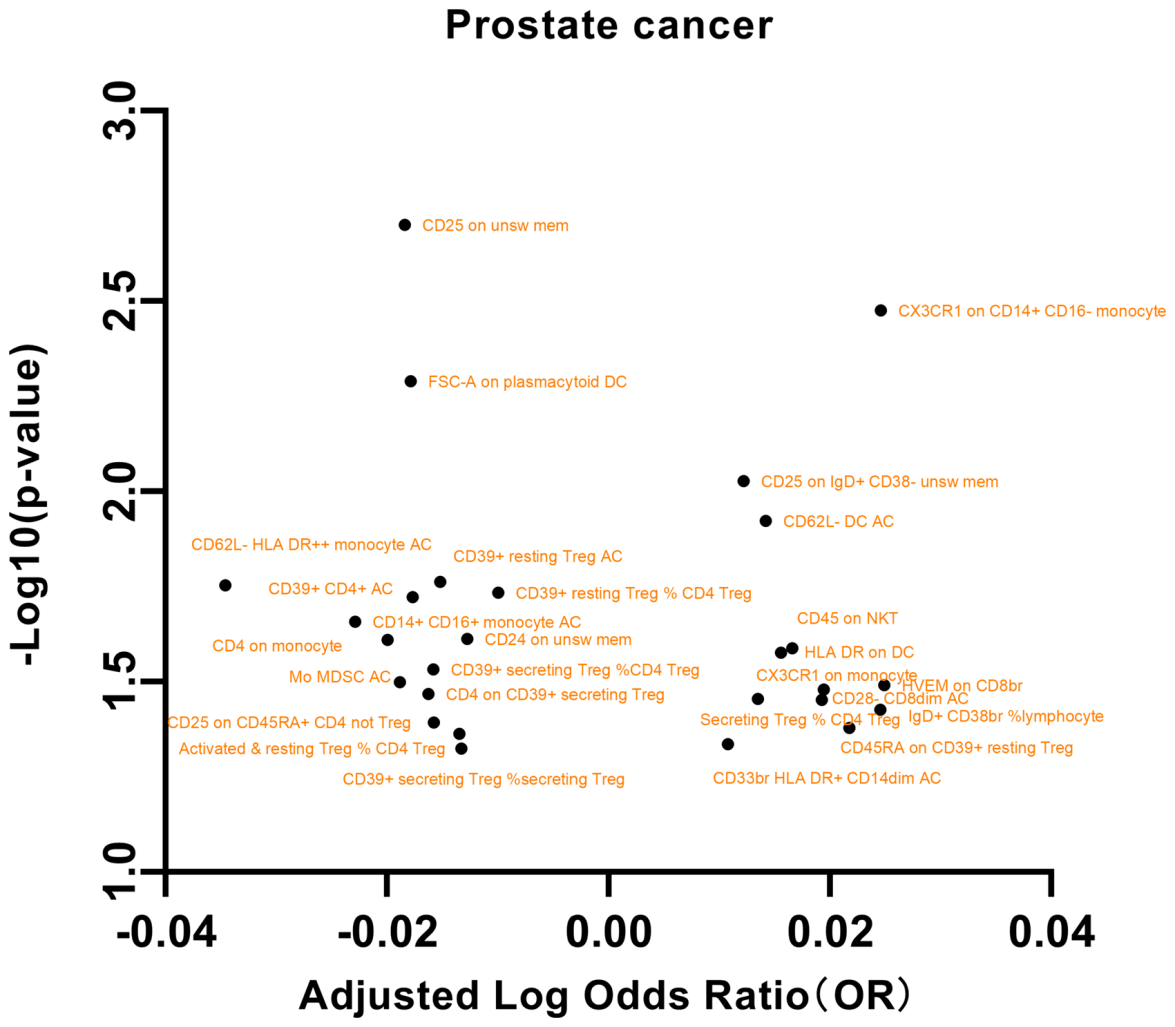
A volcano plot of the causal effects of gut microbes on prostate cancer.

We initially selected 731 immune phenotypes for analysis of their impact on PCa (Supplementary Table S5). Our examination of the relationships between these immune phenotypes and PCa revealed several significant associations (Figure 3). In particular, we observed that the maturation stages of T cells, specifically HVEM on CD8br (OR = 1.0589, 95% CI = 1.0048 to 1.11602, *p* = 0.0322), were associated with increased susceptibility to PCa. In B cells, the presence of CD25 on IgD+ CD38- unswitched memory cells (OR = 1.0285, 95% CI = 1.0069 to 1.0505, *p* = 0.0093), as well as the percentage of IgD+ CD38br lymphocytes (OR = 1.0581, 95% CI = 1.0033 to 1.1159, *p* = 0.0373), were found to be correlated with an increased risk of PCa. Conversely, the presence of CD25 on unswitched memory cells (OR = 0.9585, 95% CI = 0.9332 to 0.9846, *p* = 0.0019) and CD24 on unswitched memory cells (OR = 0.9710, 95% CI = 0.9465 to 0.9962, *p* = 0.0243) was associated with a decreased risk of PCa. Additional noteworthy associations were observed with exposures such as dendritic cells, regulatory T (Treg) cells, and myeloid cells. These findings underscore the intricate associations between prostate cancer and diverse cellular markers. However, our examination of pleiotropy and heterogeneity did not yield significant findings. Similarly, in the reverse MR analysis, no significant results were identified (Supplementary Table S6).

#### 3.2.3 Mediation analysis

In this study, both the intestinal microbiota and immune cells were found to have causal relationships with prostate diseases. The gut microbiota appears to mediate the pathway between immune cells and prostate diseases. Based on the results from the analysis of immune cells on outcomes, we screened gut microbiota immune cells as mediators. We initially selected one intestinal microbial condition with the smallest *p* value for mediation, but as some intestinal microbial conditions with the smallest *p* values failed to identify specific immune cell mediators, we continued screening with the second smallest p value. Our results indicated that the class Mollicutes inhibited the role of Secreting Treg % CD4+ Treg, Activated & resting Treg % CD4 Treg, and Mo MDSC AC in reducing the risk of PCa (Table 1).

**Table 1:** In the context of mediation analysis, betaall is used to represent the overall effect from exposure to outcome, beta1 is employed to signify the direct effect from exposure to mediator, beta2 is utilized to denote the direct effect from mediator to outcome, beta12 is calculated as the product of beta1 and beta2 and represents the estimated mediated effect, while beta12_p indicates the *p* value associated with the mediated effect.

Prostate disease	Gut microbiota	Immune cell	method	nsnp	b	se	pval	lo_ci	up_ci	or	or_lci95	or_uci95	beta_all	beta_dir	beta1	beta12	beta12_p	beta2	lci	lci_p	p	se	uci	uci-p	z
Prostate cancer	class.Mollicutes. id.3920	Secreting Treg % CD4 Treg	MR Egger	12	0.0674	0.3197	0.8374	−0.5592	0.6939	1.0697	0.5717	2.0015	−0.2717	−0.2800	0.2687	0.0083	−0.0307	0.0310	−0.0495	0.1820	0.7775	0.0295	0.0661	−0.2434	0.2826
		Activated & resting Treg % CD4 Treg	Weighted median	12	−0.2109	0.1148	0.0661	−0.4358	0.0140	0.8098	0.6467	1.0141													
		Mo MDSC AC	Inverse variance weighted	12	−0.2717	0.0951	0.0043	−0.4581	−0.0852	0.7621	0.6325	0.9183													
			Simple mode	12	−0.3018	0.1772	0.1166	−0.6491	0.0456	0.7395	0.5225	1.0466													
			Weighted mode	12	−0.2523	0.1577	0.1378	−0.5613	0.0567	0.7770	0.5705	1.0583													
Prostatitis	genus.Eubacteriu mnodatumgroup .id.11297	CD8 on EM CD8br	MR Egger	11	0.5372	1.4081	0.7117	−2.2227	3.2971	1.7112	0.1083	27.0344	0.6500	0.7277	−0.2464	−0.0778	−0.1196	0.3156	−0.1746	−0.2687	0.1156	0.0494	0.0191	0.0294	−1.5735
			Weighted median	11	0.5953	0.4162	0.1526	−0.2205	1.4111	1.8136	0.8022	4.1006													
			Inverse variance weighted	11	0.6500	0.3038	0.0324	0.0545	1.2455	1.9155	1.0560	3.4745													
			Simple mode	11	1.0180	0.6817	0.1662	−0.3181	2.3542	2.7678	0.7275	10.5292													
			Weighted mode	11	0.7593	0.6981	0.3023	−0.6090	2.1276	2.1367	0.5439	8.3944													
Benign Prostatic Hyperplasia	genus.Dorea.id. 1997	CD28- CD25++ CD8br AC	MR Egger	12	−0.0151	0.2926	0.9598	−0.5887	0.5584	0.9850	0.5551	1.7479	−0.2889	−0.2726	0.3355	−0.0163	0.0565	−0.0486	−0.1194	0.4133	0.7564	0.0526	0.0868	−0.3004	−0.3102
		CD16-CD56 on HLA DR+ NK	Weighted median	12	−0.2899	0.1394	0.0376	−0.5632	−0.0166	0.7484	0.5694	0.9836													
			Inverse variance weighted	12	−0.2889	0.0999	0.0038	−0.4847	−0.0932	0.7491	0.6159	0.9110													
			Simple mode	12	−0.3019	0.2494	0.2515	−0.7908	0.1870	0.7394	0.4535	1.2056													
			Weighted mode	12	−0.3101	0.2297	0.2041	−0.7603	0.1400	0.7334	0.4675	1.1503													

### 3.3 Prostatitis

#### 3.3.1 Associations between the gut microbiota and prostatitis

**Figure 4:**
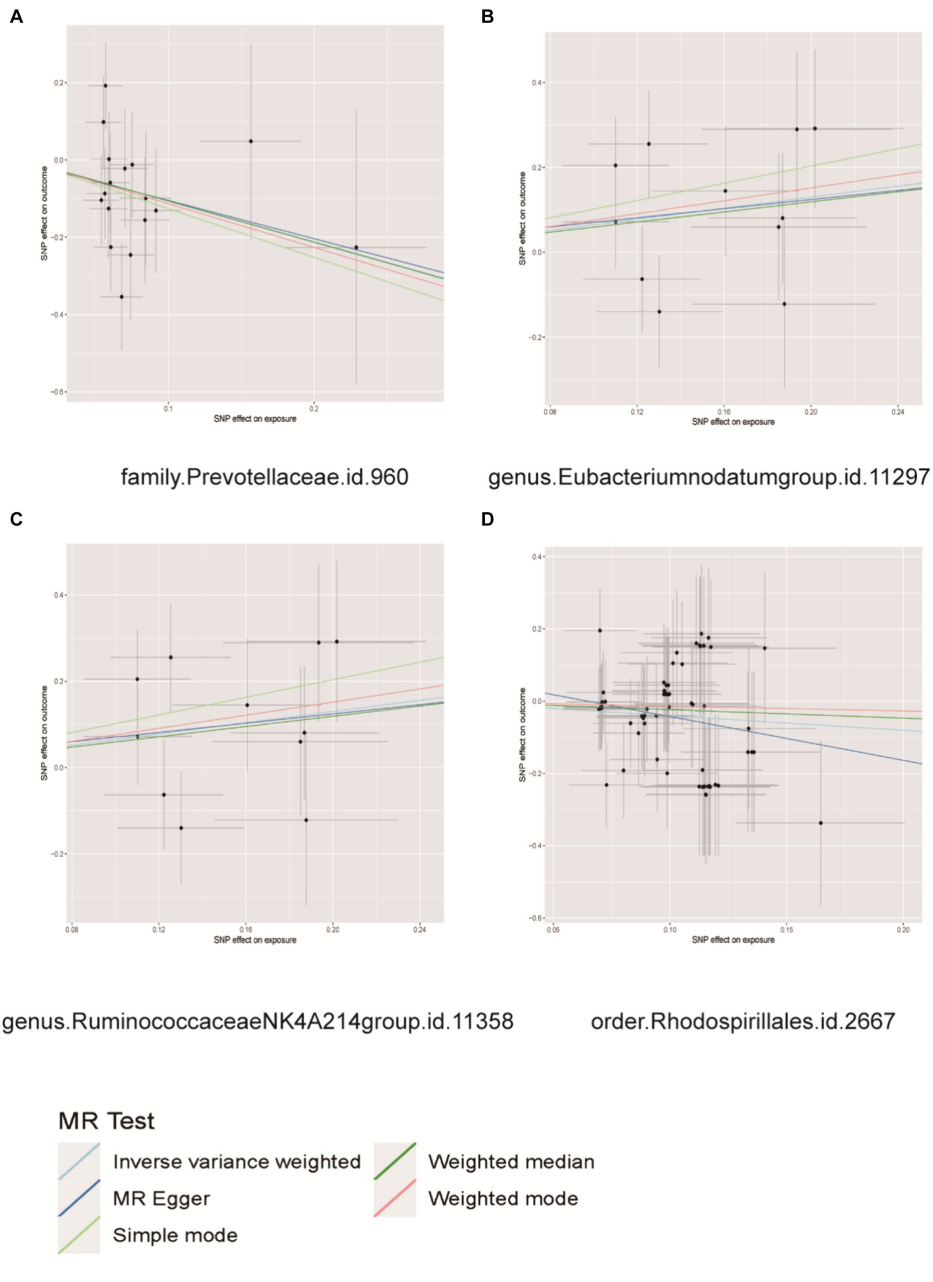
Scatter plots for causal effects of gut microbes on prostatitis. **(A)** Family Prevotellaceae, **(B)** genus *Eubacterium nodatum* group, **(C)** genus Ruminococcaceae NK4A214 group, **(D)** order Rhodospirillales.

According to the results of MR analysis (Figure 4), there exists an association between the genetic susceptibility of the *Eubacterium nodatum* group and the Ruminococcaceae NK4A214 group, both of which are intestinal microbial groups, and an elevated risk of PCa. The abundance of the genera *Eubacterium nodatum* (OR = 1.9154, 95% CI = 1.0560 to 3.4745, *p* = 0.0324) and Ruminococcaceae NK4A214 group (OR = 1.9154, 95% CI = 1.0560 to 3.4745, *p* = 0.0324) was significantly increased in PCa patients. The genetic predisposition to two intestinal microbial taxa, the family Prevotellaceae and the order Rhodospirillales, was associated with a decreased risk of prostatitis. The family Prevotellaceae (OR = 0.3439, 95% CI = 0.1357 to 0.8715, *p* = 0.0244) and order Rhodospirillales (OR = 0.6693, 95% CI = 0.4543 to 0.9861, *p* = 0.0042) were significantly associated with a decreased risk of prostatitis (Supplementary Table S3). Importantly, our results indicated heterogeneity and pleiotropy, and the results of sensitivity tests such as MR–Egger and WM supported the main findings, showing consistent results (Supplementary Figures S4–S6). No significant correlations were observed in the reverse MR analysis, as presented in Supplementary Table S4.

#### 3.3.2 Associations between immune cells and prostatitis

**Figure 5:**
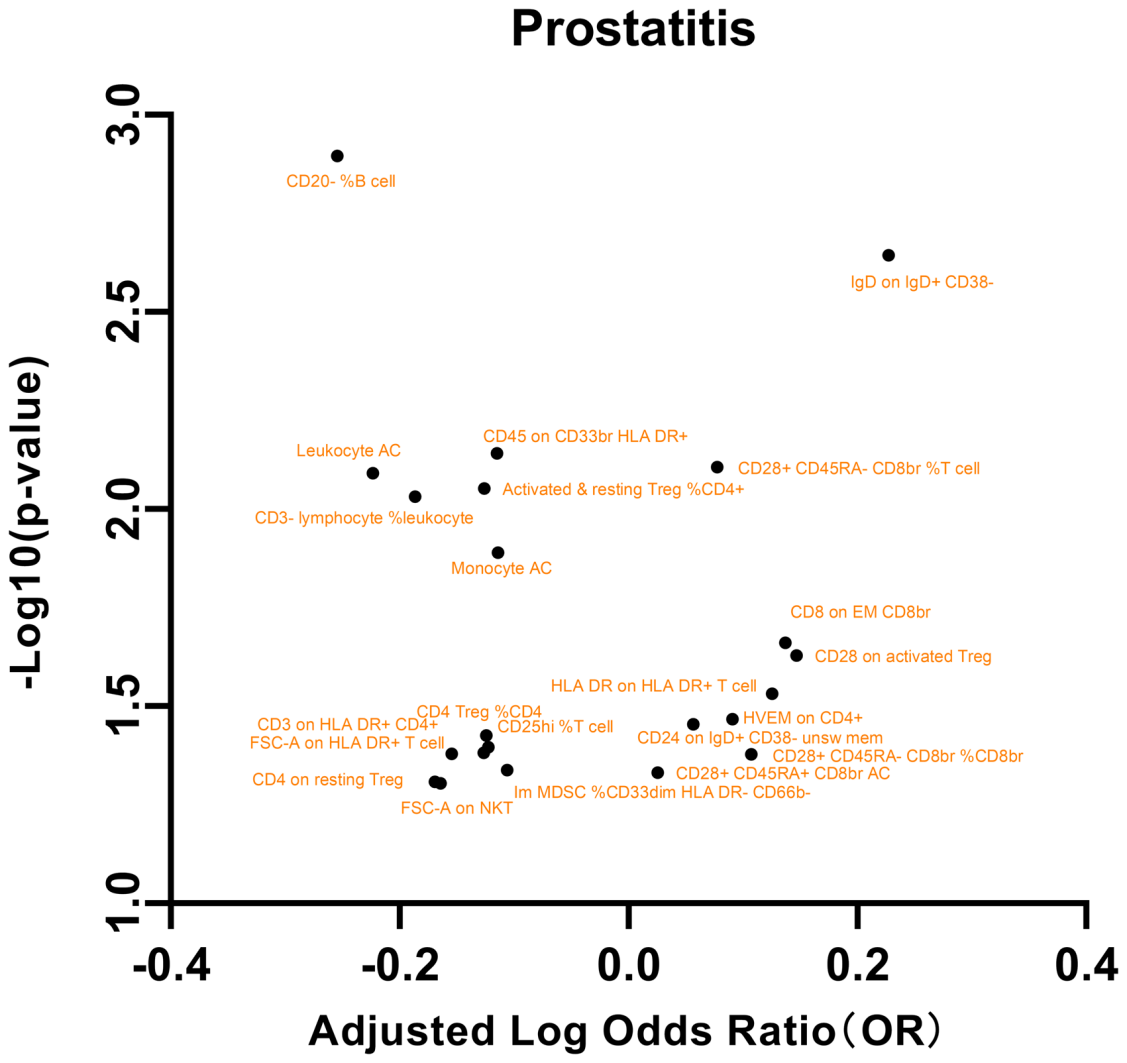
A volcano plot of the causal effects of gut microbes on prostatitis.

We subsequently analyzed the correlation between these immune phenotypes and prostatitis, as shown in Figure 5. The maturation stages of T cells, specifically the expression of HVEM on CD4+ cells, were significantly associated with an elevated risk of prostatitis (OR = 1.2328, 95% CI = 1.0157 to 1.4963; *p* = 0.0341). Similarly, in B cells, the presence of CD24 on IgD+ CD38- unsw mem cells (OR = 1.2797, 95% CI = 1.0090 to 1.6231, *p* = 0.0419) and IgD on IgD+ CD38- cells (OR = 1.6871, 95% CI = 1.2058 to 2.3605, *p* = 0.0022) has also been shown to be associated with an increased risk of prostatitis. In contrast, a lower percentage of CD20- B cells (OR = 0.5568, 95% CI = 0.3900 to 0.7950, *p* = 0.0012) was correlated with a reduced risk of prostatitis (Supplementary Table S5). Additionally, significant associations were observed with other exposures, such as TBNK cells, Treg cells, and myeloid cells. These results highlight the intricate connections between specific cellular markers and prostatitis. No significant results were found in the analysis of pleiotropy or heterogeneity. In addition, no statistically significant findings were detected in the reverse MR analysis (Supplementary Table S6).

#### 3.3.3 Mediation analysis

The presence of the *Eubacterium nodatum* group genus in instances of prostatitis might hinder the activity of CD8 on EM CD8br immune cells, leading to prostatitis (Table 1).

### 3.4 Benign prostatic hyperplasia

#### 3.4.1 Associations between the gut microbiota and BPH

**Figure 6:**
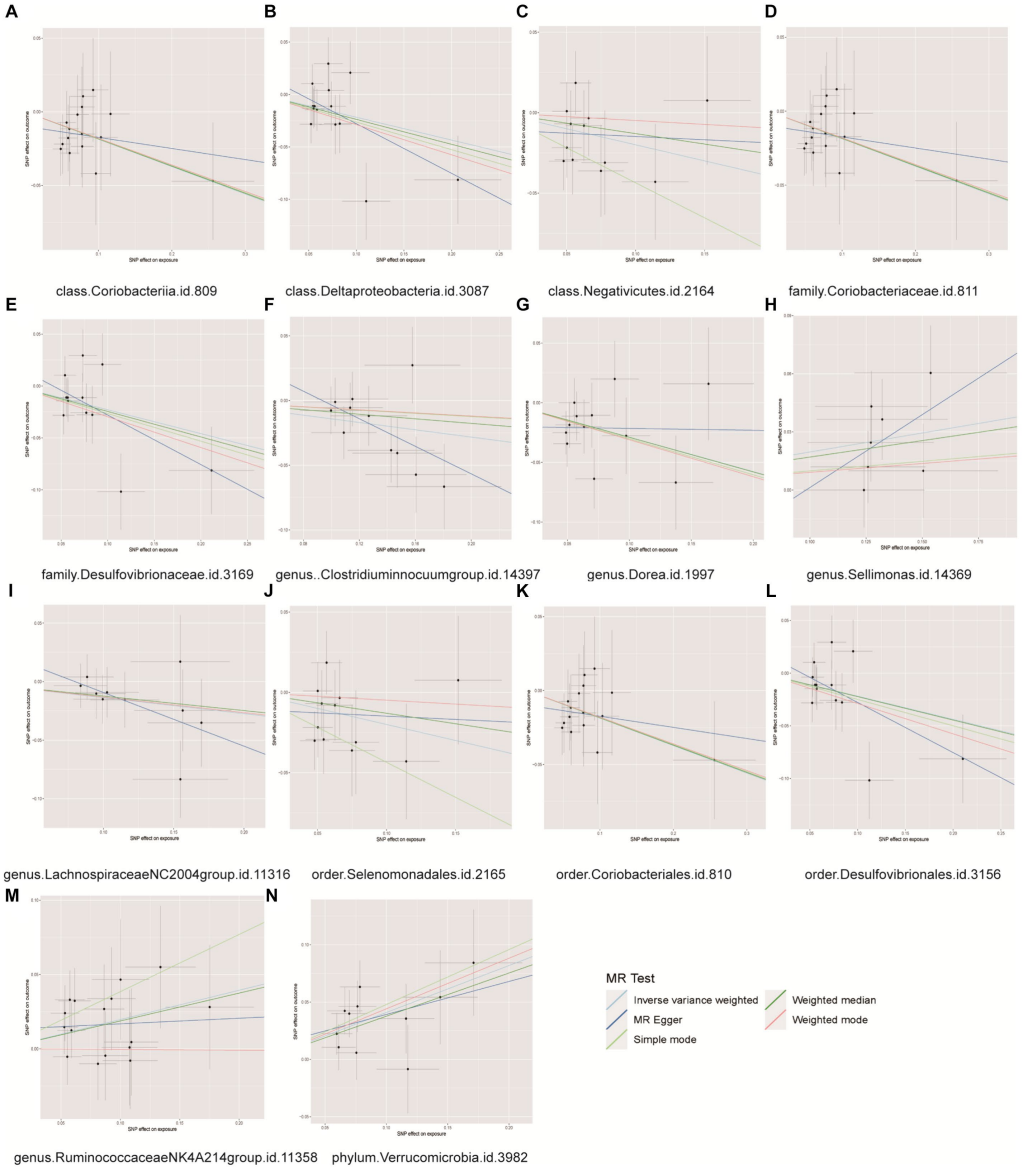
Scatter plots for causal effects of gut microbes on benign prostatic hyperplasia. **(A)** Class Coriobacteriia, **(B)** class Deltaproteobacyeria, **(C)** class Negativicutes, **(D)** family Coriobacteriaceae, **(E)** family Desulfovibrionaceae, **(F)** genus Clostridiuminnocuumgroup, **(G)** genus Dorea, **(H)** genus Sellimonas, **(I)** genus LachnospiraceaeNC2004group, **(J)** order Selenomonadales, **(K)** order Coriobacteriales, **(L)** order Desulfovibrionales, **(M)** genus RuminococcaceaeNK4A214group, **(N)** phylum Verrucomicrobia.

The MR analysis depicted in Figure 6 demonstrated that individuals with a genetic predisposition to three specific intestinal microbial taxa (genus Sellimonas, genus Ruminococcaceae NK4A214 group, phylum Verrucomicrobia) exhibited an elevated susceptibility to BPH. The abundances of the genera Sellimonas (OR=1.1253, 95% CI= 1.009~1.2550, *p*=0.0339), Ruminococcaceae NK4A214 group (OR=1.2176, 95% CI=1.0369~1.4299, *p*=0.0162), and phylum Verrucomicrobia (OR=1.2176, 95% CI=1.0369 ~ 1.4299, *p*=0.0162) significantly increased in the BPH group. The genetic predispositions of 11 intestinal microbial taxa, namely, the class Coriobacteriia, class Deltaproteobacteria, class Negativicutes, family Coriobacteriaceae, family Desulfovibrionaceae, genus *Clostridium innocuum* group, genus Dorea, genus Lachnospiraceae NC2004 group, order Coriobacteriales, order Desulfovibrionales, and order Selenomonadales, were found to be correlated with a reduced likelihood of developing BPH. The classes Coriobacteriia (OR=0.8302, 95% CI=0.7076–0.9739, *p*=0.0223), Deltaproteobacteria (OR=0.8046, 95% CI=0.6778–0.9551, *p*=0.01297), and Negativicutes (OR=0.8188, 95% CI=0.6706–0.9998, *p*=0.0498), as well as the family Coriobacteriaceae (OR=0.8302, 95% CI=0.7076–0.9739, *p*=0.0223), family Desulfovibrionaceae (OR = 0.7949, 95% CI = 0.6566–0.9623, *p* = 0.0185), genus *Clostridium innocuum* group (OR = 0.8683, 95% CI = 0.7801–0.9665, *p* = 0.0097), genus Dorea (OR = 0.7490, 95% CI = 0.6158–0.9110, *p* = 0.0038), genus Lachnospiraceae NC2004 group (OR = 0.8719, 95% CI = 0.7607–0.9993, *p* = 0.0489), order Coriobacteriales (OR = 0.8302, 95% CI = 0.7076–0.9739, *p* = 0.0223), order Desulfovibrionales (OR = 0.8030, 95% CI = 0.6710–0.9610, *p* = 0.0166), and the order Selenomonadales (OR = 0.8188, 95% CI = 0.6706–0.9998, *p* = 0.0498), were correlated with a reduced likelihood of developing BPH (Supplementary Table S3). Importantly, our results indicated heterogeneity and pleiotropy, and sensitivity tests such as MR–Egger and WM supported the main findings, showing consistent results (Supplementary Figures S7–S9). No significant correlations were found in the reverse MR analysis (Supplementary Table S4).

#### 3.4.2 Associations between immune cells and BPH

**Figure 7:**
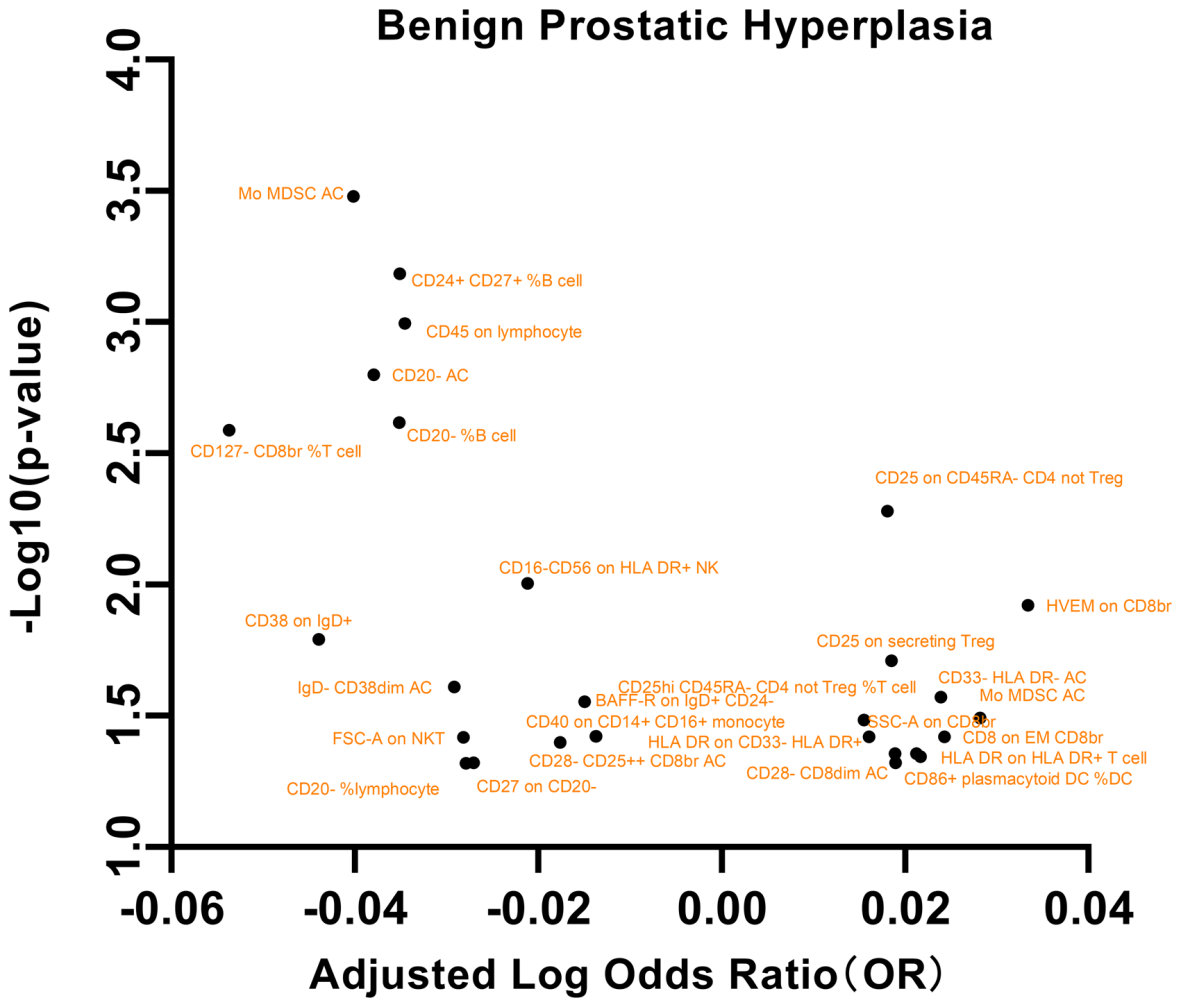
A volcano plot of the causal effects of gut microbes on benign prostatic hyperplasia.

Our analysis of the relationships between these immune phenotypes and BPH revealed several significant associations (Figure 7). This study revealed a significant association between the maturation stage of T cells, specifically HVEM on CD8br (OR = 1.0799, 95% CI = 1.0170 to 1.1467, *p* = 0.0119), and an elevated risk of BPH. Additionally, an increased risk of BPH was also observed for Treg cells, specifically CD25hi CD45RA- CD4 not Treg %T cell (OR = 1.0364, 95% CI = 1.0029~1.0710, *p* = 0.0328), Lymphocyte AC (OR = 1.0671, 95% CI = 1.0055~1.1325, *p* = 0.0322), CD25 on CD45RA- CD4 not Treg (OR = 1.0424, 95% CI = 1.0124~1.0733, *p* = 0.0052), and CD25 on secreting Treg (OR = 1.0435, 95% CI = 1.0069~1.0816, *p* = 0.0194). This study revealed an inverse correlation between CD28- CD25++ CD8br AC (OR = 0.9603, 95% CI = 0.9239 ~ 0.9981, *p* = 0.0399) and CD127- CD8br %T cell (OR = 0.8837, 95% CI = 0.8154 ~ 0.9576, *p* = 0.0025) and a decreased risk of BPH. Additional significant associations were observed with factors such as TBNK cells, the maturation stage of T cells, and myeloid cells. These findings underscore the intricate correlation between specific cellular markers and BPH. No significant findings were detected in the analysis of pleiotropy or heterogeneity. In addition, no statistically significant findings were detected in the reverse MR analysis, as indicated in Supplementary Table S6.

#### 3.4.3 Mediation analysis

Interestingly, in BPH, the genus Dorea promoted the role of CD28- CD25++ CD8br AC and CD16-CD56 on HLA DR+ NK cells in reducing the risk of BPH (Table 1).

## 4 Discussion

**Figure 8:**
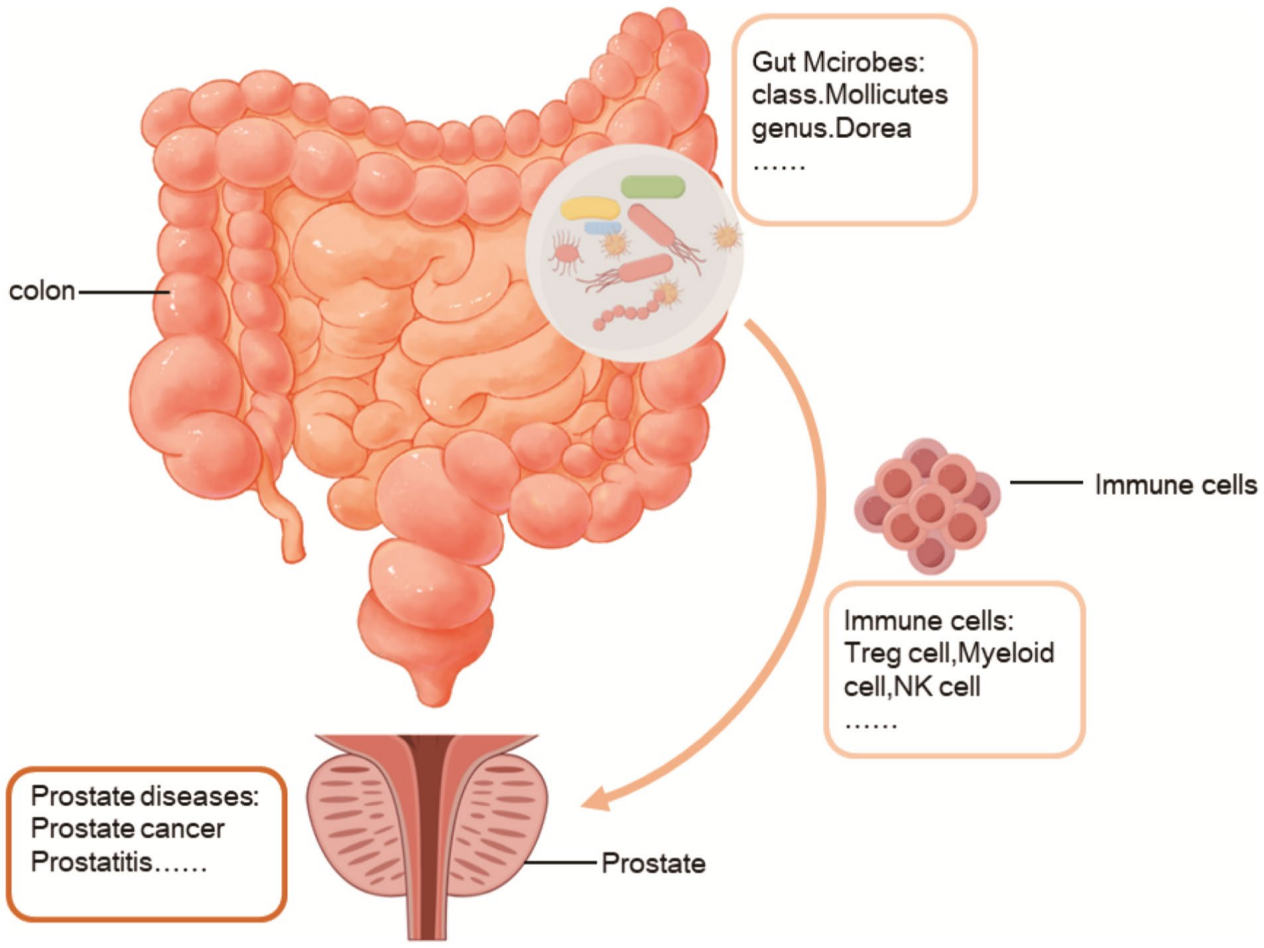
Gut microbes influence the development of prostate diseases through immune cells.

The objective of this study was to investigate the impact of gut microbiota on prostate diseases, aiming to elucidate its potential therapeutic benefits or detrimental effects. Here, for the first time, we utilized gut microbiota as mediators between the immune cells and prostate diseases. The gut microbiota is a complex and ever-changing system that is impacted by several variables. Simultaneously, as stated in the literature, the disruption of the gut microbiota’s ecological equilibrium arises from the imbalance between the gut microbiome and the intestinal epithelium. Intestinal epithelial cells have a crucial function in preserving the mutually beneficial interaction between the gut microbiota and the host. They do this by creating a protective layer of mucus, releasing different immunological substances, and conveying bacterial antigens (Shi et al., 2017; Okumura and Takeda, 2017; Liu et al., 2020). On the other hand, our research findings indicate that alterations in the gut microbiota can impact the maturation of immune cells, specifically B cells, Treg cells, Myeloid cells, and Classical dendritic cells, thereby affecting prostate diseases (Figure 8). These findings align with the studies conducted by Ji-Eun Kim, Leonie Brockmann, Baichao Yu, and other scholars (Kim et al., 2022; Brockmann et al., 2023; Yu et al., 2021). Using mediation analysis, we revealed that various immune cell subsets, including Secreting Treg % CD4 Treg, Activated & resting Treg % CD4 Treg, Mo MDSC AC, CD8 on EM CD8br, CD28- CD25++ CD8br AC, and CD16-CD56 on HLA DR+ NK, exert both protective and detrimental effects on prostate diseases by modulating the abundance of specific bacterial taxa, such as class Mollicutes, genus *Eubacterium nodatum* group and genus Dorea.

While cancer has traditionally been regarded as a genetic disorder, emerging research indicates a potential association between the microbiome and cancer. Studies have demonstrated variations in gut microbiota profiles among individuals with PCa and BPH, with PCa patients exhibiting elevated levels of *Bacteroides massiliensis* compared with those with BPH (Golombos et al., 2018). Our findings revealed a correlation between the presence of Akkermansia and Butyrivibrio genera and susceptibility to PCa. Previous research findings have indicated that the levels of Akkermansia in the gut microbiota of PCa patients undergoing targeted androgen axis therapy, as well as those treated with abiraterone for castration-resistant PCa (CRPC), are impacted (Tsai et al., 2022; Daisley et al., 2020). The influence of bacteria on susceptibility to PCa is likely complex and involves specific microbial functions, host reactions, and broader interactions within the microbiome (Fujita et al., 2022). Therapeutic approaches utilizing the immune system to target cancer cells have yielded diverse prognostic results for various solid tumors and blood-related cancers. Immunotherapy plays a pivotal role in the management of PCa, particularly in the treatment of CRPC (Cha et al., 2020).

Our research revealed a notable inverse relationship between CD24/CD25 expression on naive mature B cells and susceptibility to PCa. This discovery is consistent with the findings of Hao (Hao et al., 2024). Our study results revealed a negative correlation between CD25-expressing regulatory T cells and genetic susceptibility to PCa in Treg cells. Additionally, our results provide further support for the notion that targeting Treg cells with anti-CD25 antibodies may have beneficial effects on the immune response against tumors (Villanueva, 2021; Lee et al., 2022). Conversely, the percentages of certain cell types, including secreting Treg % CD4 Treg and CD28- CD8dim AC, were positively correlated with the risk of PCa. Furthermore, myeloid cells and monocytes, along with other immune cells, display diverse risk associations with PCa. These findings underscore the intricate role of immune cells in the development and progression of PCa.

The connection between gut microbiota and prostatitis is complex, as evidenced by prior research demonstrating a significant reduction in gut microbiota diversity in individuals with chronic prostatitis (Shoskes et al., 2016). The present study findings substantiated the relationship between four gut microbiota taxa (family Prevotellaceae, genus *Eubacterium nodatum* group, genus Ruminococcaceae NK4A214 group, and order Rhodospirillales) and susceptibility to prostatitis. Previous studies have established a correlation between dysregulated gut microbiota composition and depressive-like behavior in mice afflicted with experimental autoimmune prostatitis (EAP). The findings of subsequent investigations revealed that the gut microbiota can modulate short-chain fatty acid production, thereby impacting Th17/Treg cell differentiation in mice with EAP (Du et al., 2022). This finding is further supported by findings from an EAP model in which hyperactivation of Th1 and Th17 cells was observed (Zhan et al., 2020; Chen et al., 2022; Murphy et al., 2015). Alternatively, Th1 cells can exert their effects by migrating to the prostate site through the expression of CXCR3 (Breser et al., 2013; Yue et al., 2023). Our study findings revealed a positive correlation between myeloid cells and susceptibility to prostatitis, while CD4 Treg %CD4, Activated & resting Treg % CD4+, and CD25hi %T cell in Treg cells were identified as suppressors of this risk. These findings suggest that Treg cells play a protective role in prostatitis patients. Additionally, our results indicated that in prostatitis, CD8 on EM CD8br may impede the increased risk posed by the genus *Eubacterium nodatum* group.

Recent research has shown that changes in the gut microbiota significantly impact the pathogenesis, progression, diagnosis, and management of BPH. Specifically, the presence of Lactobacillus, Flavonifractor, and Acetatifactor in the BPH model has been linked to key indicators of this condition (An et al., 2023). Xia established a causal relationship between gut microbiota and BPH via MR analysis. These findings revealed significant associations between BPH and the presence of Eisenbergiella, Ruminococcaceae (UCG009), and Escherichia shigella (Xia et al., 2023). The findings of this study revealed that the Ruminococcaceae NK4A214 group, Sellimonas genus, and Verrucomicrobia phylum did not exhibit a protective effect on BPH. Further investigation and confirmation are required to elucidate the mechanisms through which gut microbiota influences the pathogenesis of BPH. Chronic inflammation in BPH can perpetually stimulate the prostate gland, thereby impacting the onset and progression of BPH (Nickel et al., 2017). M2 macrophages are the predominant inflammatory cells that infiltrate and proliferate within the prostate gland. The secretion of cytokines and growth factors plays a crucial role in driving the pathogenesis of BPH (Gandaglia et al., 2017). CD8+ T cells are commonly found in the periglandular region surrounding the epithelial tubes, whereas lymphoid aggregates consisting of B lymphocytes and follicular T lymphocytes are located within the fibromuscular stroma (De Nunzio et al., 2011). BPH cells can augment the inflammatory response by recruiting additional inflammatory cells through diverse mechanisms (Cao et al., 2022). Our study findings suggested that B cells play a protective role in the pathogenesis of BPH. Conversely, there is an intricate interplay between Treg cells, myeloid cells, and TBNK cells and BPH. Our findings demonstrate that in BPH, certain subsets of immune cells, such as CD28- CD25++ CD8br antigen-presenting cells and CD16-CD56 natural killer cells expressing HLA DR+, contribute to a reduced risk of colonization by the genus Dorea. However, further investigation is required to identify how gut microbiota modulate immune cell responses and subsequently impact BPH.

This study has several limitations. The investigation focused exclusively on the impact of gut microbiota on prostate diseases in the presence of immune cell mediation, potentially overlooking biases arising from other variables. Currently, we merely depend on the GWAS database and do not incorporate additional population data in order to enhance the general applicability of our findings. Furthermore, individual-level variations were not considered for a more in-depth examination of the relationships among gut microbiota, immune cells, and prostate diseases. Finally, because MR analysis depends on unverifiable assumptions, more experimental and clinical evidence is needed to determine how gut microbiota affect prostate disorders through immune cells.

## 5 Conclusion

This study highlights the complex relationships among the gut microbiota, immune cells and prostate diseases. The involvement of the gut microbiota in regulating immune cells to impact prostate diseases could provide novel methods and concepts for its therapy and management.

## Data availability statement

The original contributions presented in the study are included in the article/Supplementary material, further inquiries can be directed to the corresponding author/s.

## Author contributions

S-YY: Writing – review & editing, Writing – original draft, Visualization, Validation, Supervision, Software, Resources, Project administration, Methodology, Investigation, Funding acquisition, Formal analysis, Data curation, Conceptualization. W-YL: Writing – review & editing, Writing – original draft, Software, Investigation, Conceptualization. SX: Writing – review & editing, Writing – original draft, Data curation, Conceptualization. X-XB: Writing – review & editing, Formal analysis, Data curation, Conceptualization. W-LX: Writing – review & editing, Formal analysis, Data curation. XW: Writing – review & editing, Software, Investigation. H-KD: Writing – original draft, Methodology. JC: Writing – original draft, Methodology. H-XD: Writing – review & editing, Supervision. L-FX: Writing – original draft, Funding acquisition. DN: Writing – review & editing, Writing – original draft, Software, Investigation, Conceptualization. C-ZL: Writing – review & editing, Writing – original draft, Resources, Funding acquisition.

## Footnotes

^1^ https://gwas.mrcieu.ac.uk/

## Supplementary material

Supplementary Figure S1 In the study examining the causal relationship between the intestinal microbiota of 10 patients with prostate cancer, the Wald ratio was calculated for each SNP individually, while both the MR–Egger and IVW methods were employed for the combined analysis of all SNPs. **(A)** class Erysipelotrichia, **(B)** class Mollicutes, **(C)** class Verrucomicrobiae, **(D)** family Erysipelotrichaceae, **(E)** family Verrucomicrobiaceae, **(F)** genus Akkermansia, **(G)** genus Buutyrivibrio, **(H)** order Erysipelotrichales, **(I)** order Verrucomicrobiales, **(J)** phylum Tenericutes

Supplementary Figure S2 A funnel plot was generated to identify SNP heterogeneity when evaluating the potential causal relationships between 10 intestinal microbiota and prostate cancer. **(A)** class Erysipelotrichia, **(B)** class Mollicutes, **(C)** class Verrucomicrobiae, **(D)** family Erysipelotrichaceae, **(E)** family Verrucomicrobiaceae, **(F)** genus Akkermansia, **(G)** genus Buutyrivibrio, **(H)** order Erysipelotrichales, **(I)** order Verrucomicrobiales, **(J)** phylum Tenericutes

Supplementary Figure S3 A leave-one-out analysis was conducted to examine the causal effects of 10 intestinal microbiota on the development of prostate cancer. **(A)** class Erysipelotrichia, **(B)** class Mollicutes, **(C)** class Verrucomicrobiae, **(D)** family Erysipelotrichaceae, **(E)** family Verrucomicrobiaceae, **(F)** genus Akkermansia, **(G)** genus Buutyrivibrio, **(H)** order Erysipelotrichales, **(I)** order Verrucomicrobiales, **(J)** phylum Tenericutes

Supplementary Figure S4 To assess the causal impact of 4 intestinal microbiota on prostatitis, the Wald ratio was applied to each SNP individually, while both the MR–Egger and IVW methods were utilized when considering all SNPs collectively. Note: (A) family Prevotellaceae, (B) genus *Eubacterium nodatum* group, (C) genus Ruminococcaceae NK4A214 group, (D) order Rhodospirillales

Supplementary Figure S5 A funnel plot was generated to identify SNP heterogeneity in evaluating the causal impacts of 4 intestinal microbiota on prostatitis. **(A)** family Prevotellaceae, **(B)** genus *Eubacterium nodatum* group, **(C)** genus Ruminococcaceae NK4A214 group, **(D)** order Rhodospirillales

Supplementary Figure S6 A leave-one-out analysis was conducted to examine the causal effects of 4 intestinal microbiota on prostatitis. **(A)** family Prevotellaceae, **(B)** genus *Eubacterium nodatum* group, **(C)** genus Ruminococcaceae NK4A214 group, **(D)** order Rhodospirillales

Supplementary Figure S7 To assess the causal effects of 14 intestinal microbiota on BPH, the Wald ratio was applied to each SNP individually, while both MR–Egger and IVW methods were utilized for the analysis of all SNPs collectively. **(A)** class Coriobacteriia, **(B)** class Deltaproteobacyeria, **(C)** class Negativicutes, **(D)** family Coriobacteriaceae, **(E)** family Desulfovibrionaceae, **(F)** genus Clostridiuminnocuumgroup, **(G)** genus Dorea, **(H)** genus Sellimonas, **(I)** genus LachnospiraceaeNC2004group, **(J)** order Selenomonadales, **(Q)** order Coriobacteriales, **(M)** order Desulfovibrionales, **(N)** genus RuminococcaceaeNK4A214group, **(O)** phylum Verrucomicrobia

Supplementary Figure S8 A funnel plot was generated to identify SNP heterogeneity in the evaluation of the causal impact of 14 intestinal microbiota on BPH. **(A)** class Coriobacteriia, **(B)** class Deltaproteobacyeria, **(C)** class Negativicutes, **(D)** family Coriobacteriaceae, **(E)** family Desulfovibrionaceae, **(F)** genus Clostridiuminnocuumgroup, **(G)** genus Dorea, **(H)** genus Sellimonas, **(I)** genus LachnospiraceaeNC2004group, **(J)** order Selenomonadales, **(Q)** order Coriobacteriales, **(M)** order Desulfovibrionales, **(N)** genus RuminococcaceaeNK4A214group, **(O)** phylum Verrucomicrobia

Supplementary Figure S9 A leave-one-out analysis was conducted to examine the causal effects of 14 intestinal microbiota on BPH. **(A)** class Coriobacteriia, **(B)** class Deltaproteobacyeria, **(C)** class Negativicutes, **(D)** family Coriobacteriaceae, **(E)** family Desulfovibrionaceae, **(F)** genus Clostridiuminnocuumgroup, **(G)** genus Dorea, **(H)** genus Sellimonas, (I) genus LachnospiraceaeNC2004group, **(J)** order Selenomonadales, **(Q)** order Coriobacteriales, **(M)** order Desulfovibrionales, **(N)** genus RuminococcaceaeNK4A214group, **(O)** phylum Verrucomicrobia

Supplementary Table S1 1650 SNPs for 75 immune cells.

Supplementary Table S2 412 SNPs for 28 gut microbiota taxa.

Supplementary Table S3 Causal effect of gut microbiota on prostate diseases.

Supplementary Table S4 Causal analysis of prostate diseases on gut microbiota.

Supplementary Table S5 Causal effect of immune cells on prostate diseases.

Supplementary Table S6 Causal analysis of prostate diseases on immune cells.
