# Supplementary figures and images for: Causality investigation among gut microbiota, immune cells, and prostate diseases: a Mendelian randomization study
Source: Front Microbiol. 2024 Sep 11;15:1445304. doi: 10.3389/fmicb.2024.1445304 (PMC11422081; doi:10.3389/fmicb.2024.1445304)

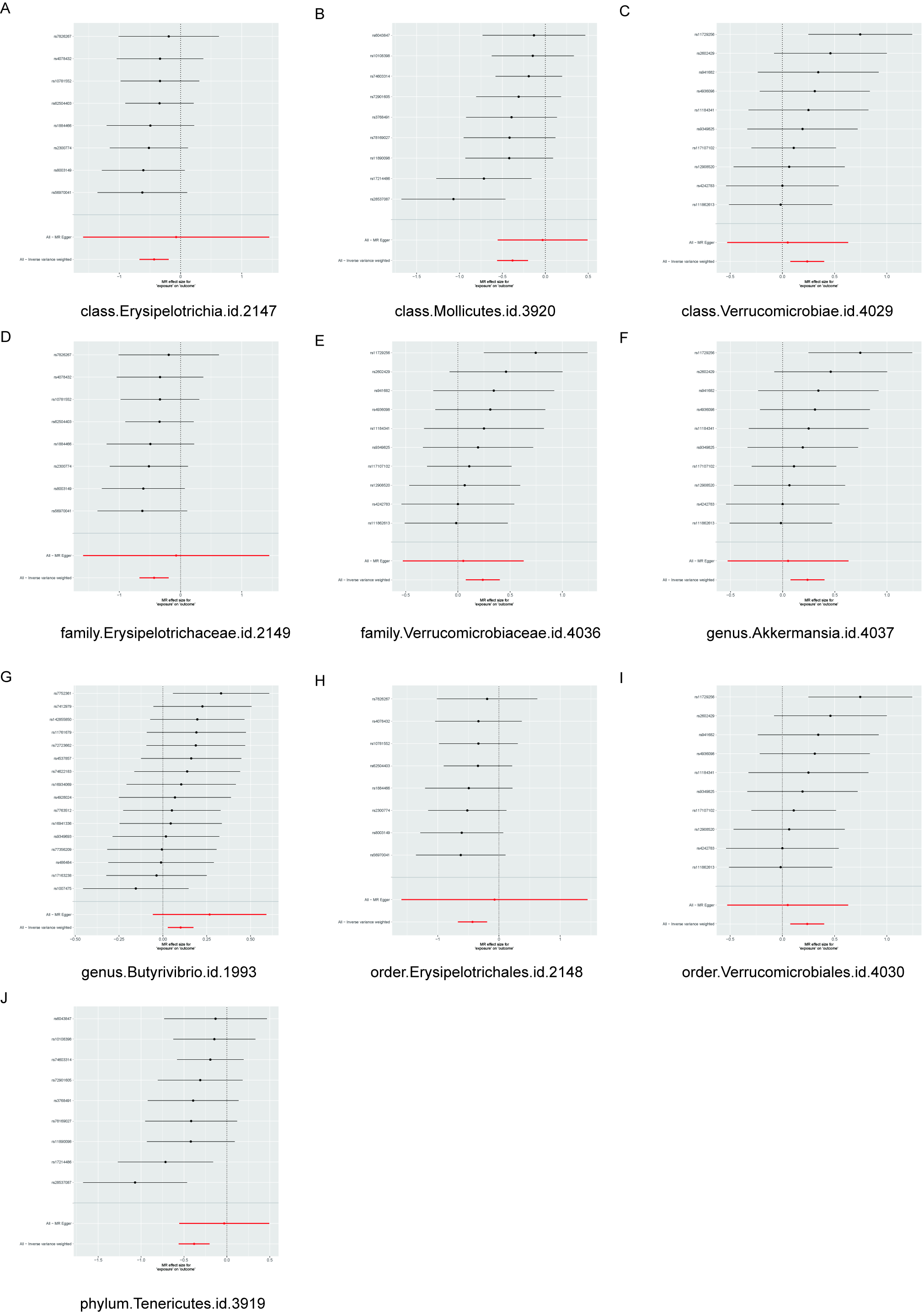

Supplement: Supplementary Figure S1 — In the study examining the causal relationship between the intestinal microbiota of 10 patients with prostate cancer, the Wald ratio was calculated for each SNP individually, while both the MR–Egger and IVW methods were employed for the combined analysis of all SNPs. (A) class Erysipelotrichia, (B) class Mollicutes, (C) class Verrucomicrobiae, (D) family Erysipelotrichaceae, (E) family Verrucomicrobiaceae, (F) genus Akkermansia, (G) genus Buutyrivibrio, (H) order Erysipelotrichales, (I) order Verrucomicrobiales, (J) phylum Tenericutes [file Image_1.TIF]

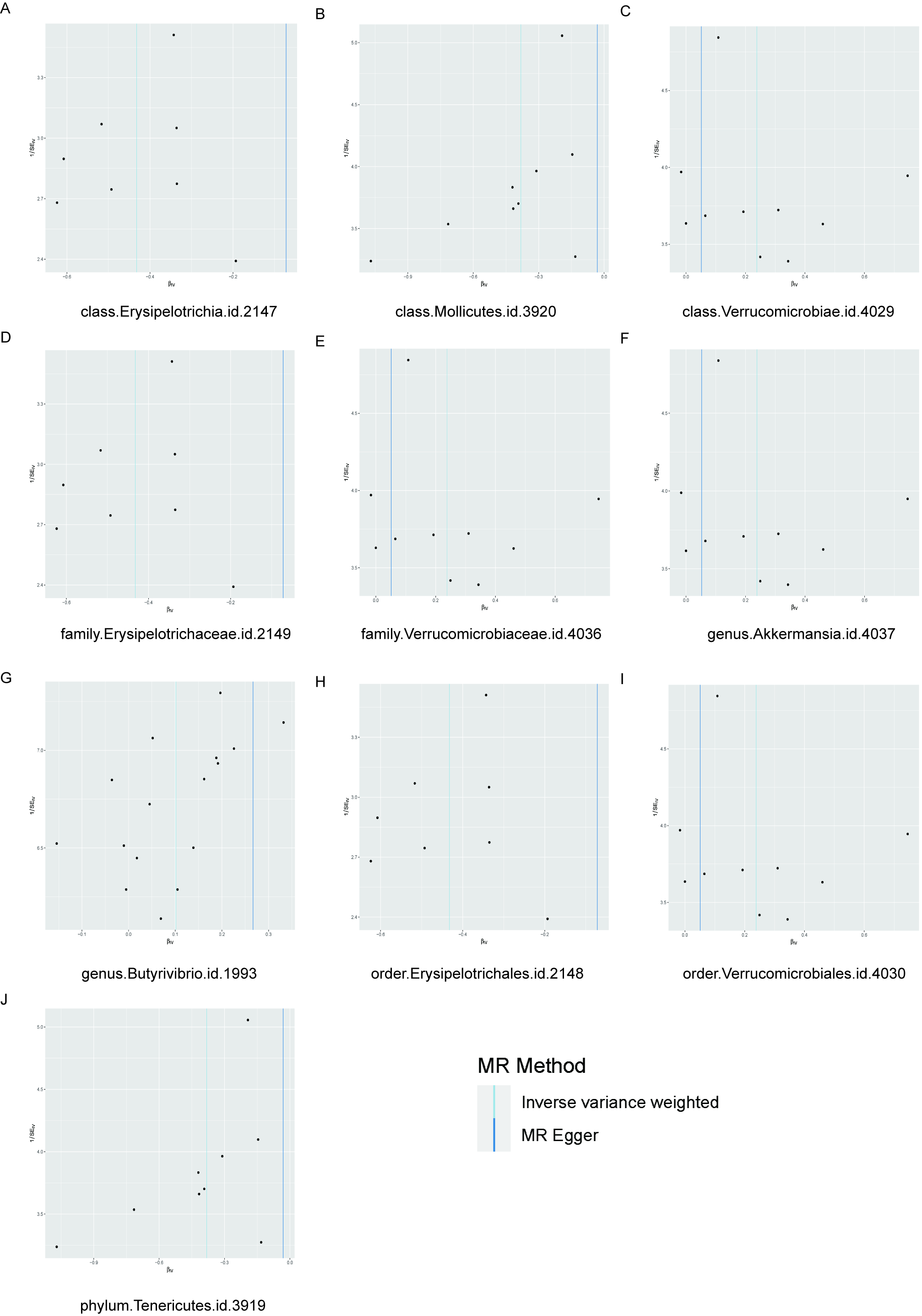

Supplement: Supplementary Figure S2 — A funnel plot was generated to identify SNP heterogeneity when evaluating the potential causal relationships between 10 intestinal microbiota and prostate cancer. (A) class Erysipelotrichia, (B) class Mollicutes, (C) class Verrucomicrobiae, (D) family Erysipelotrichaceae, (E) family Verrucomicrobiaceae, (F) genus Akkermansia, (G) genus Buutyrivibrio, (H) order Erysipelotrichales, (I) order Verrucomicrobiales, (J) phylum Tenericutes [file Image_2.TIF]

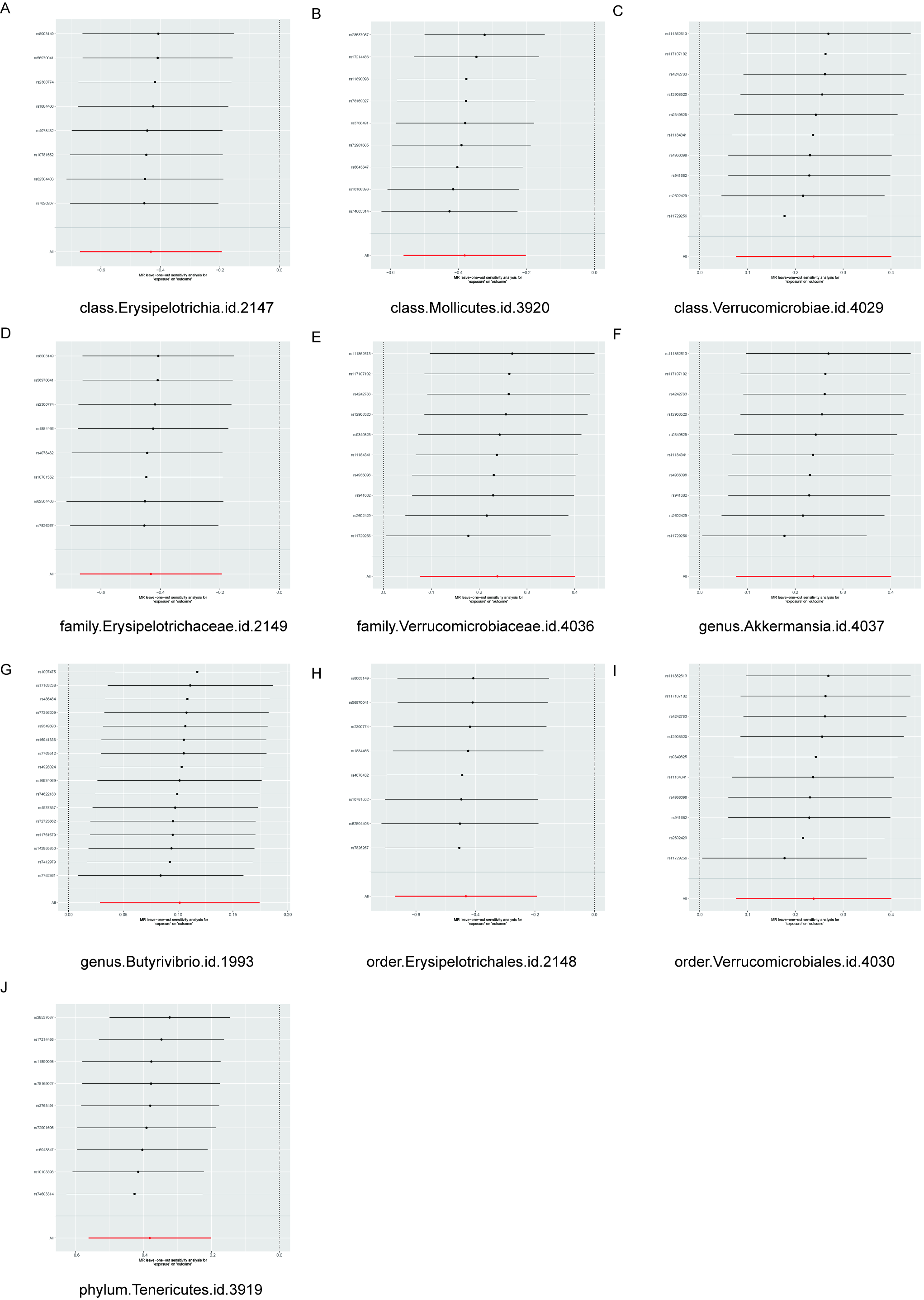

Supplement: Supplementary Figure S3 — A leave-one-out analysis was conducted to examine the causal effects of 10 intestinal microbiota on the development of prostate cancer. (A) class Erysipelotrichia, (B) class Mollicutes, (C) class Verrucomicrobiae, (D) family Erysipelotrichaceae, (E) family Verrucomicrobiaceae, (F) genus Akkermansia, (G) genus Buutyrivibrio, (H) order Erysipelotrichales, (I) order Verrucomicrobiales, (J) phylum Tenericutes [file Image_3.TIF]

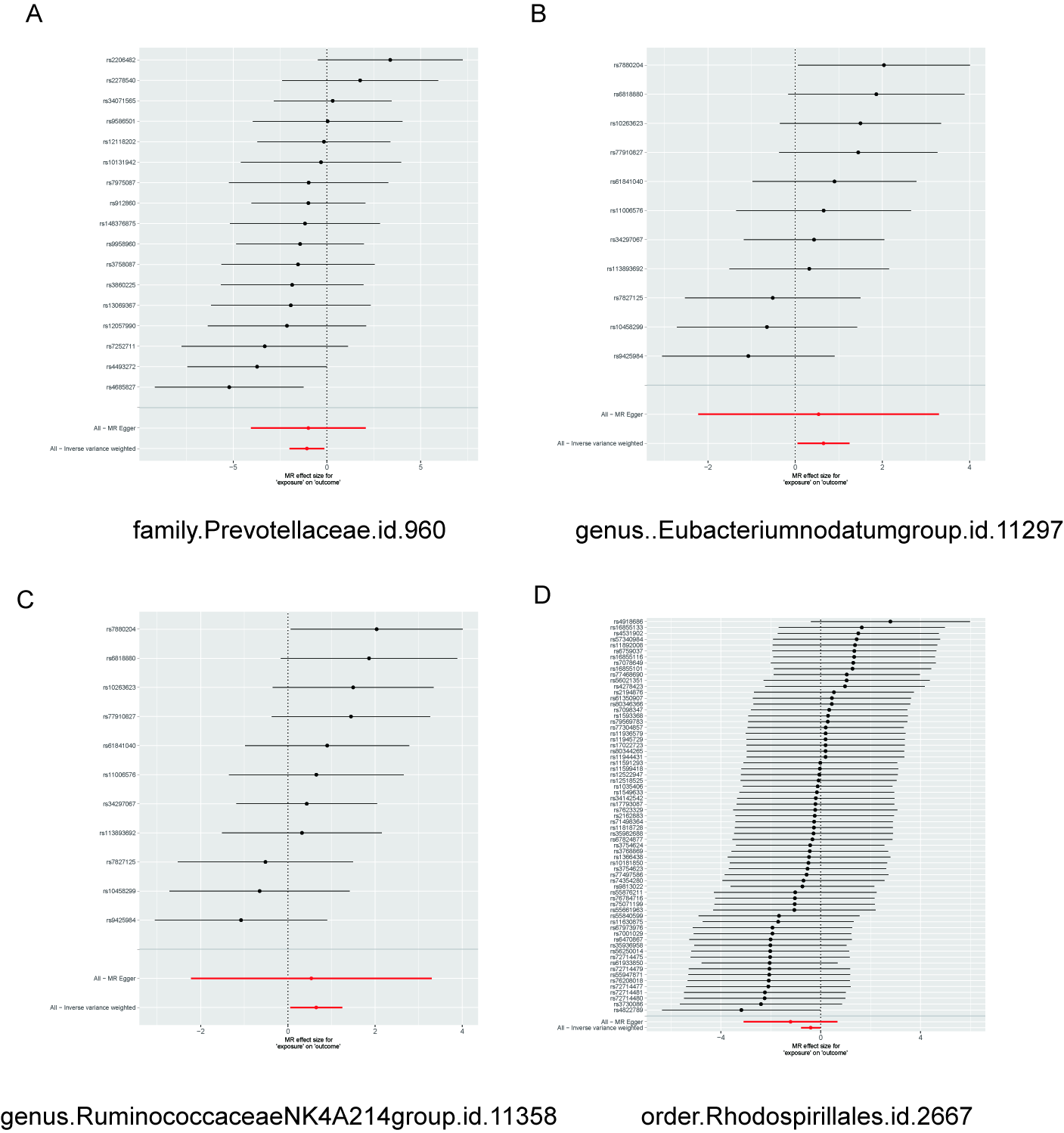

Supplement: Supplementary Figure S4 — To assess the causal impact of 4 intestinal microbiota on prostatitis, the Wald ratio was applied to each SNP individually, while both the MR–Egger and IVW methods were utilized when considering all SNPs collectively. Note: (A) family Prevotellaceae, (B) genus Eubacterium nodatum group, (C) genus Ruminococcaceae NK4A214 group, (D) order Rhodospirillales [file Image_4.TIF]

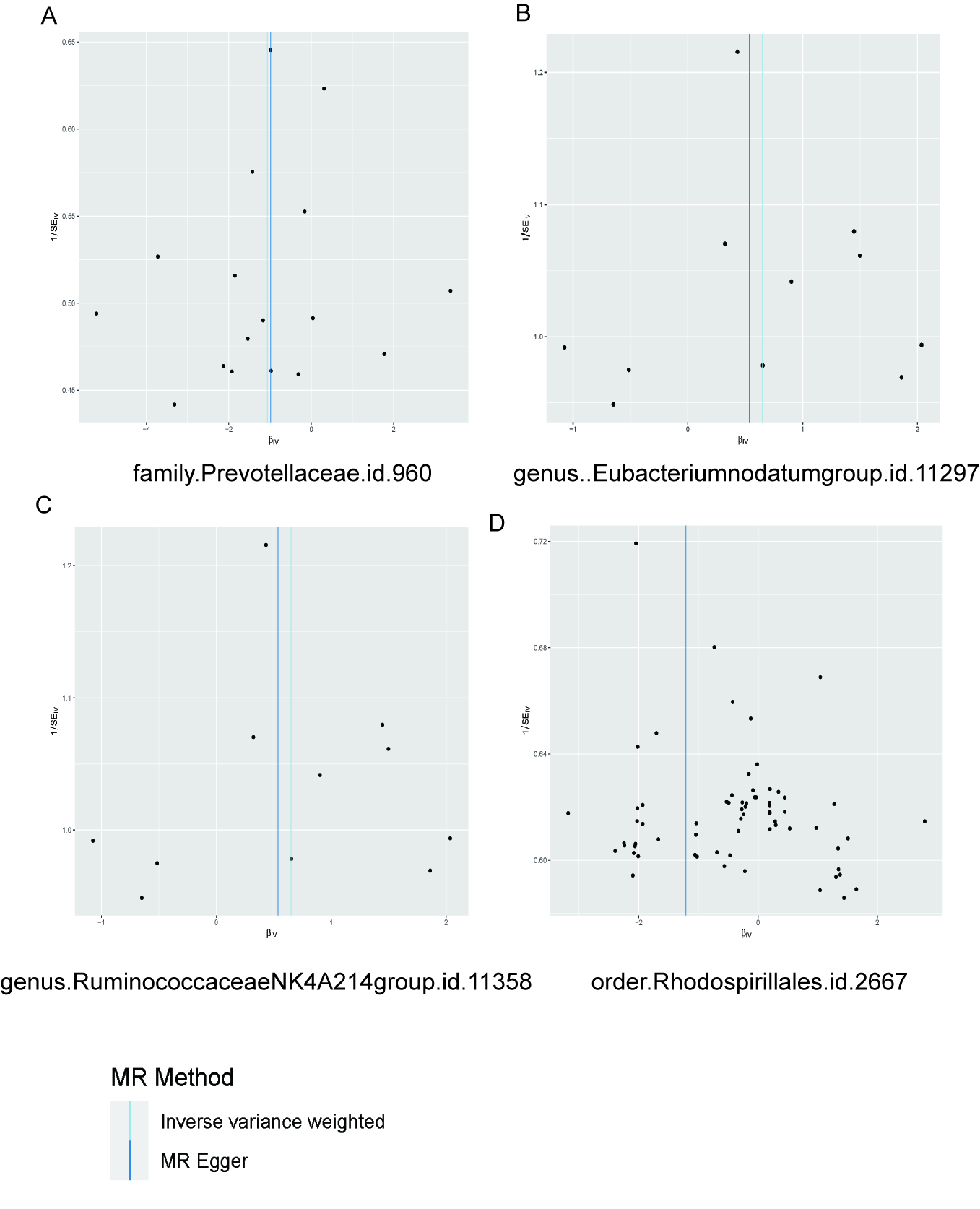

Supplement: Supplementary Figure S5 — A funnel plot was generated to identify SNP heterogeneity in evaluating the causal impacts of 4 intestinal microbiota on prostatitis. (A) family Prevotellaceae, (B) genus Eubacterium nodatum group, (C) genus Ruminococcaceae NK4A214 group, (D) order Rhodospirillales [file Image_5.TIF]

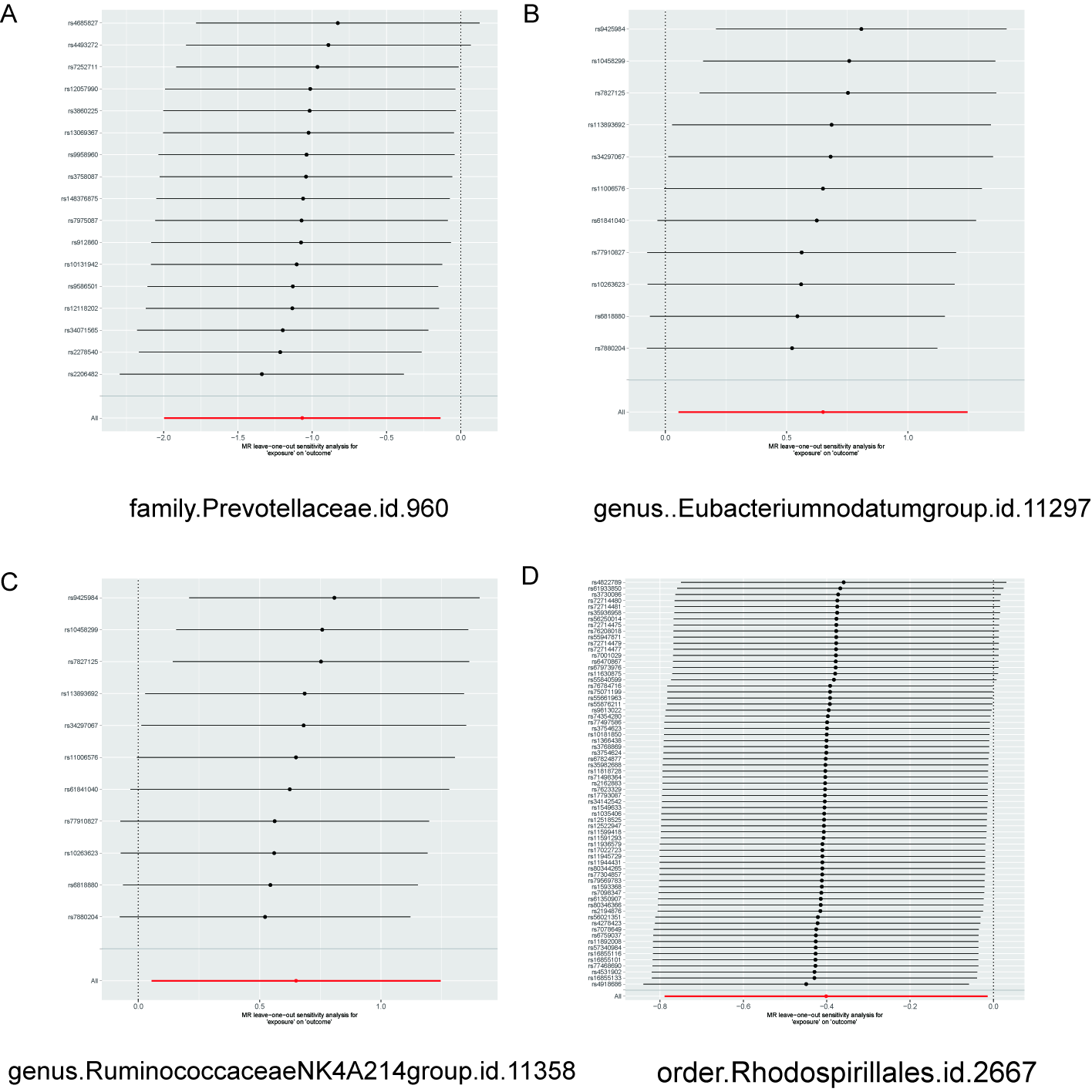

Supplement: Supplementary Figure S6 — A leave-one-out analysis was conducted to examine the causal effects of 4 intestinal microbiota on prostatitis. (A) family Prevotellaceae, (B) genus Eubacterium nodatum group, (C) genus Ruminococcaceae NK4A214 group, (D) order Rhodospirillales [file Image_6.TIF]

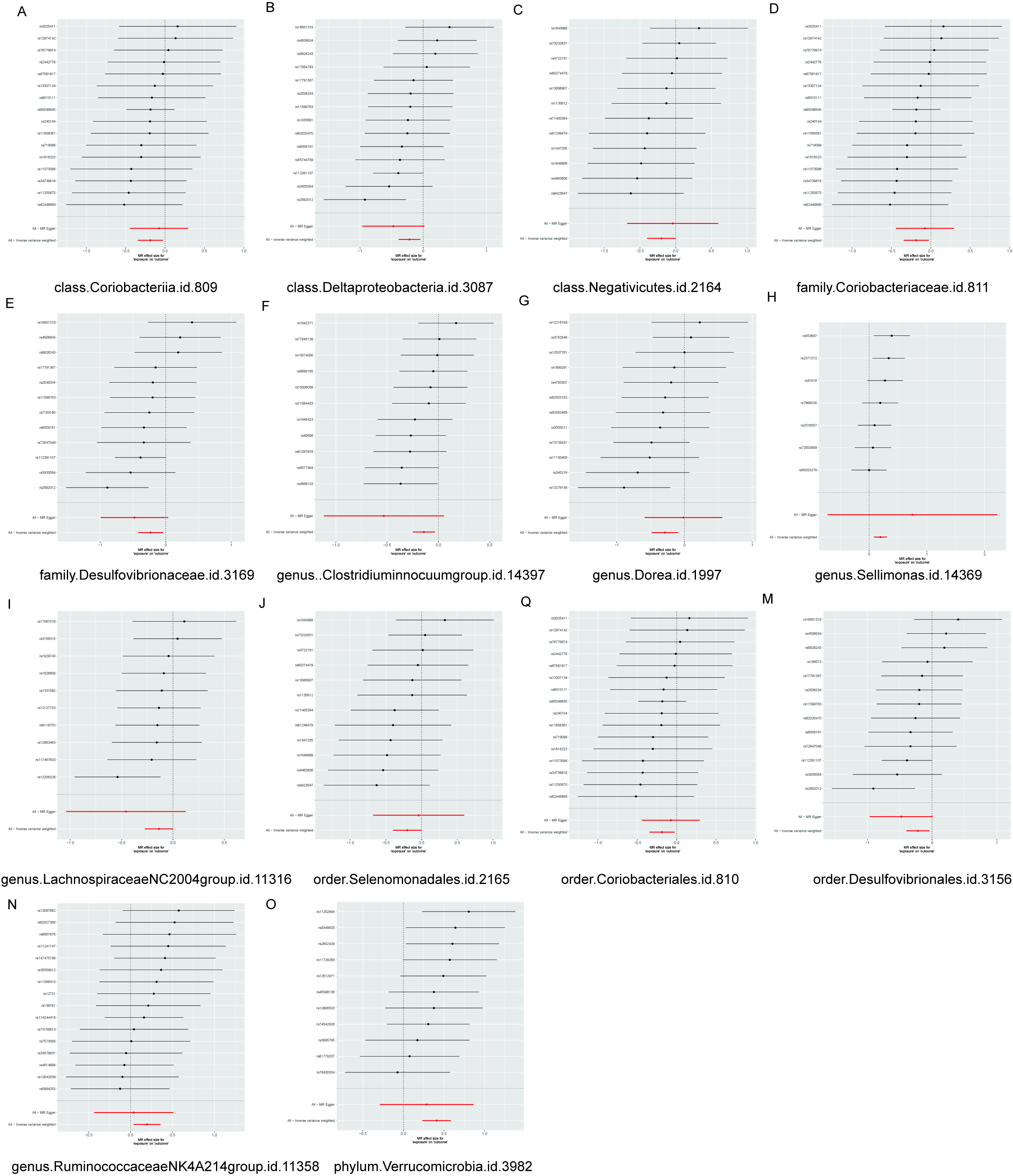

Supplement: Supplementary Figure S7 — To assess the causal effects of 14 intestinal microbiota on BPH, the Wald ratio was applied to each SNP individually, while both MR–Egger and IVW methods were utilized for the analysis of all SNPs collectively. (A) class Coriobacteriia, (B) class Deltaproteobacyeria, (C) class Negativicutes, (D) family Coriobacteriaceae, (E) family Desulfovibrionaceae, (F) genus Clostridiuminnocuumgroup, (G) genus Dorea, (H) genus Sellimonas, (I) genus LachnospiraceaeNC2004group, (J) order Selenomonadales, (Q) order Coriobacteriales, (M) order Desulfovibrionales, (N) genus RuminococcaceaeNK4A214group, (O) phylum Verrucomicrobia [file Image_7.TIF]

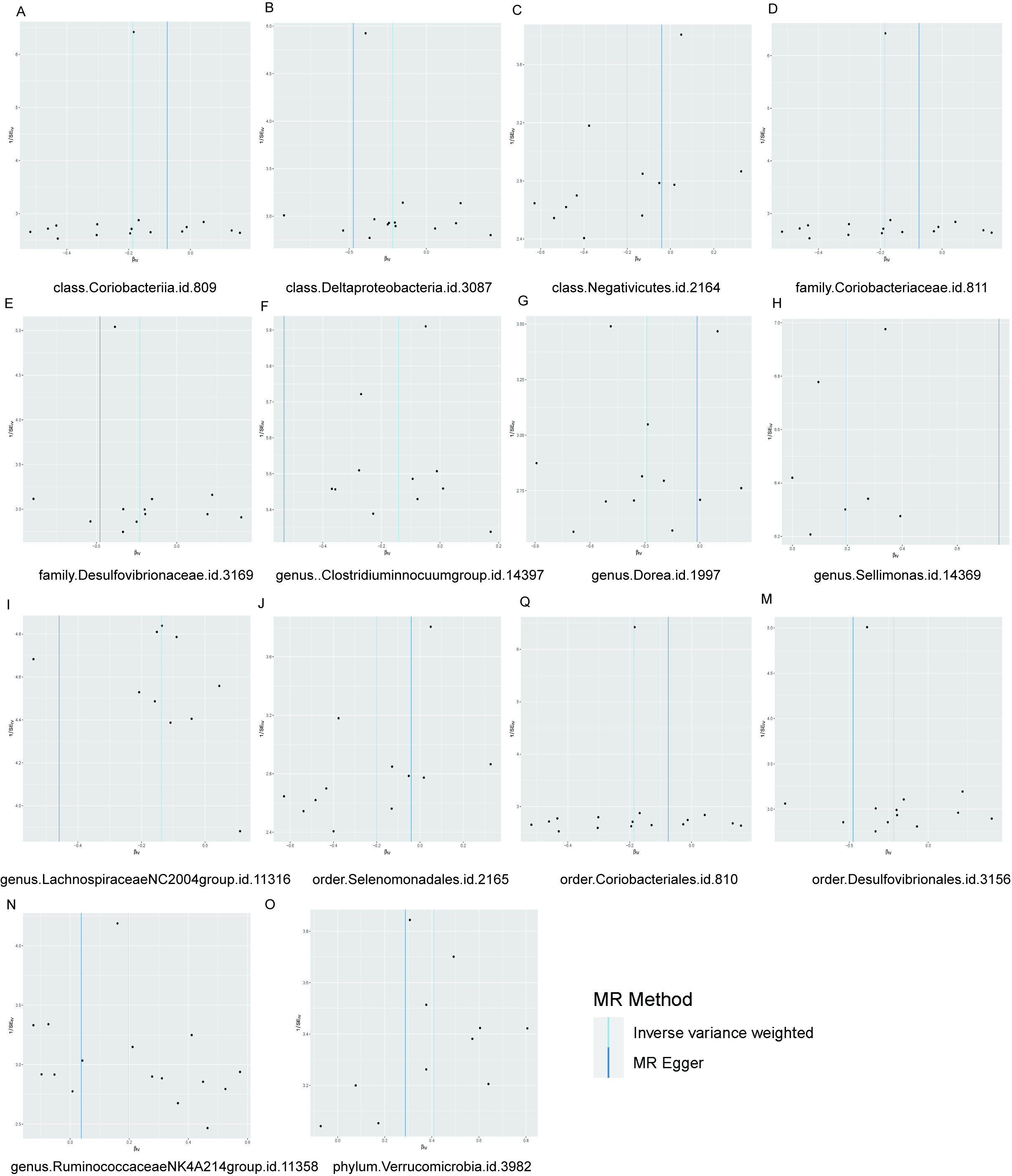

Supplement: Supplementary Figure S8 — A funnel plot was generated to identify SNP heterogeneity in the evaluation of the causal impact of 14 intestinal microbiota on BPH. (A) class Coriobacteriia, (B) class Deltaproteobacyeria, (C) class Negativicutes, (D) family Coriobacteriaceae, (E) family Desulfovibrionaceae, (F) genus Clostridiuminnocuumgroup, (G) genus Dorea, (H) genus Sellimonas, (I) genus LachnospiraceaeNC2004group, (J) order Selenomonadales, (Q) order Coriobacteriales, (M) order Desulfovibrionales, (N) genus RuminococcaceaeNK4A214group, (O) phylum Verrucomicrobia [file Image_8.TIF]

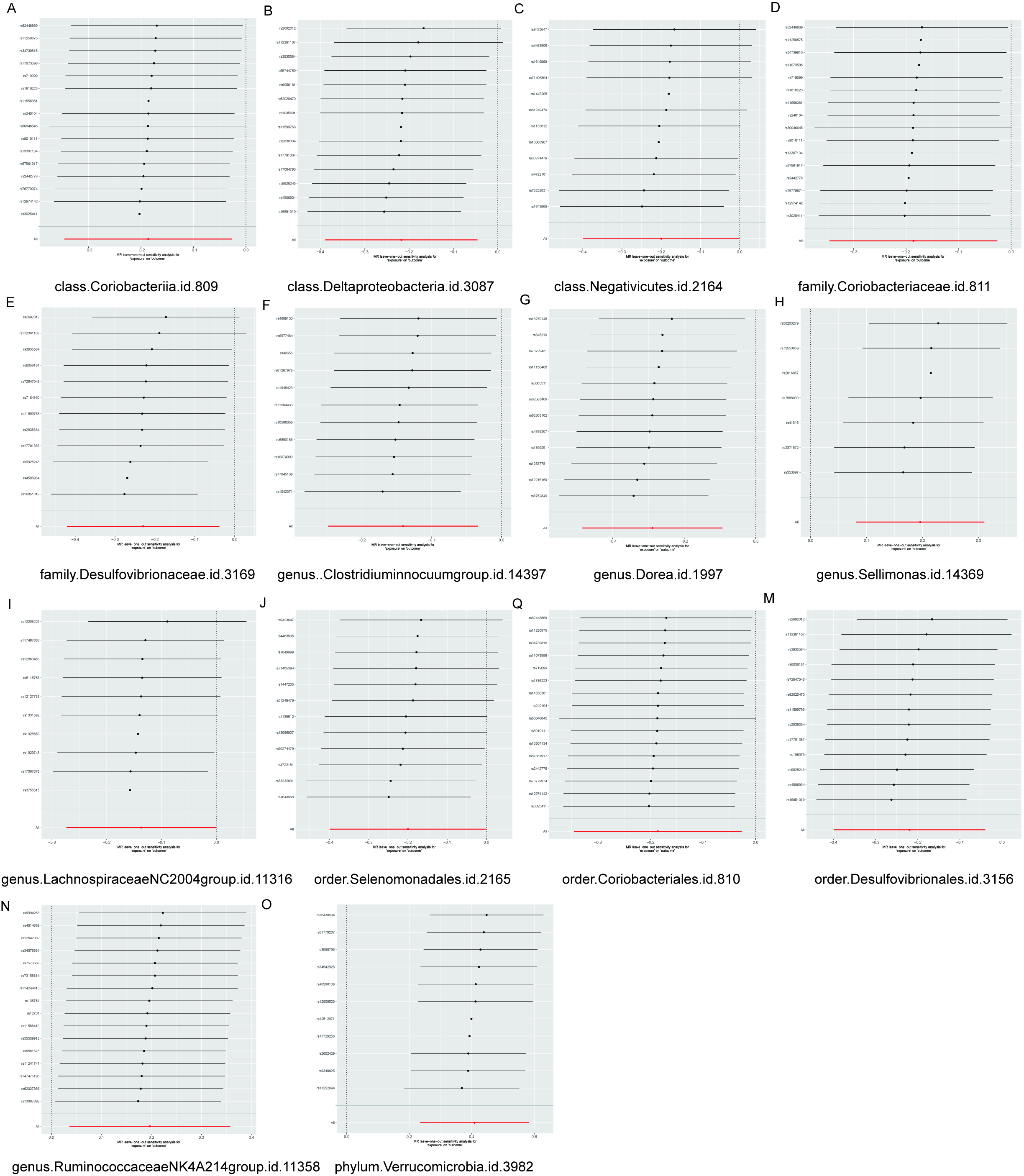

Supplement: Supplementary Figure S9 — A leave-one-out analysis was conducted to examine the causal effects of 14 intestinal microbiota on BPH. (A) class Coriobacteriia, (B) class Deltaproteobacyeria, (C) class Negativicutes, (D) family Coriobacteriaceae, (E) family Desulfovibrionaceae, (F) genus Clostridiuminnocuumgroup, (G) genus Dorea, (H) genus Sellimonas, (I) genus LachnospiraceaeNC2004group, (J) order Selenomonadales, (Q) order Coriobacteriales, (M) order Desulfovibrionales, (N) genus RuminococcaceaeNK4A214group, (O) phylum Verrucomicrobia [file Image_9.TIF]
