# Supplementary material for: Causality investigation among gut microbiota, immune cells, and prostate diseases: a Mendelian randomization study
Source: Front Microbiol. 2024 Sep 11;15:1445304. doi: 10.3389/fmicb.2024.1445304 (PMC11422081; doi:10.3389/fmicb.2024.1445304)
Supplement: Supplementary file 16 [file Presentation_1.pdf]

STROBE-MR checklist of recommended items to address in reports of Mendelian randomization studies[1 2]

Item No.	Section		Checklist item	Page No.	Relevant text from manuscript
1	**TITLE and ABSTRACT**		Indicate Mendelian randomization (MR) as the study's design in the title and/or the abstract if that is a main purpose of the study		Causality investigation among Gut Microbiota, Immune Cells, and Prostate Diseases: A Mendelian Randomization Study
	**INTRODUCTION**				
2	**Background**		Explain the scientific background and rationale for the reported study. What is the exposure? Is a potential causal relationship between exposure and outcome plausible? Justify why MR is a helpful method to address the study question		Background: The gut microbiota has been demonstrated to have a significant role in the pathogenesis and progression of a variety of diseases, including prostate cancer, prostatitis, and benign prostatic hyperplasia. Potential links between prostate diseases, immune cells and the gut microbiota have not been adequately investigated.
3	**Objectives**		State specific objectives clearly, including pre-specified causal hypotheses (if any). State that MR is a method that, under specific assumptions, intends to estimate causal effects		Potential links between prostate diseases, immune cells and the gut microbiota have not been adequately investigated.
	**METHODS**				
4	**Study design and data sources**		Present key elements of the study design early in the article. Consider including a table listing sources of data for all phases of the study. For each data source contributing to the analysis, describe the following:		
		a)	Setting: Describe the study design and the underlying population, if possible. Describe the setting, locations, and relevant dates, including periods of recruitment, exposure, follow-up, and data collection, when available.		This study encompasses three primary components, as depicted in Figure 1: the investigation of the causal effects of 196 gut microbial taxa on three categories of prostate diseases (Step 1A); the analysis of the causal effects of 731 immune cells on three types of prostate diseases (Step 2A); and the implementation of mediation analysis to examine the role of the gut microbiota in mediating the pathway from immune cells to prostate diseases (Step 3). SNPs were defined as instrumental variables (IVs)
		b)	Participants: Give the eligibility criteria, and the sources and methods of selection of participants. Report the sample size, and whether any power or sample size calculations were carried out prior to the main analysis		The summary data for prostatitis, PCa, and BPH can be found in the GWAS Catalogue under the accession numbers GCST90084104, GCST90079112, and GCST90081804, respectively. In this study, a secondary analysis of publicly

		available GWAS summary statistics was conducted, with ethical approval obtained for each original GWAS.
c)	Describe measurement, quality control and selection of genetic variants	Initially, SNPs demonstrating significant associations with the gut microbiota ($P < 1 \times 10^{-5}$) were chosen for analysis. SNPs exhibiting linkage disequilibrium (LD) were omitted, with a predetermined threshold of $P < 5 \times 10^{-6}$. The LD between the identified SNPs and the gut microbiota was required to meet the criteria of $r^2 < 0.001$ and a distance exceeding 10,000 kb (Myers et al., 2020). A crucial component of MR analysis involves verifying that the impact of an SNP on an individual's exposure aligns with the effect of the SNP on the outcome for the same allele. Following this comparison, palindromic SNPs (defined as those with A/T or G/C alleles) were subsequently excluded from the analysis.
d)	For each exposure, outcome, and other relevant variables, describe methods of assessment and diagnostic criteria for diseases	To evaluate the potential causal association between the gut microbiota and immune cells in relation to prostate diseases, we conducted independent two-sample MR analyses (steps 1A and 2A in Figure 1).The analytic methods employed were IVW, MR-Egger, weighted median, simple mode, and weighted mode, and Wald ratio tests were conducted for variables featuring only one IVW result (Pierce and Burgess 2013). In cases where discrepancies arose between these methods, the IVW results were considered the primary outcome. IVW is a method for calculating a weighted average for random variable analysis. One uses the variance of every random variable as a weight. This approach can help to lower the mean's fluctuation. SNPs are employed to simulate the genetic grouping process. This randomizing strategy allows one to overcome the usual linear character of genetic variation and other potential factors, producing more exact causal inference. The MR–Egger test was utilized to identify outliers and assess horizontal pleiotropy. By considering the similarity in causality, a weighted model technique may categorize SNPs into distinct subsets and utilize the subset with the greatest number of SNPs to evaluate the causal connection between exposure and result. Thus, funnel plots were employed to assess potential directional pleiotropy.

			To mitigate the potential inaccuracies caused by SNPs, a leave-one-out study was conducted to evaluate the impact of individual SNPs on the MR estimates. Meanwhile, we use website tools （https://gwas.mrcieu.ac.uk/） to eliminate confounding factors. All Mendelian randomization analyses were performed via the ‘Two Sample MR’ (version 0.5.6) package in R version 4.3.1, setting statistical significance at $P < 0.05$.
		e) Provide details of ethics committee approval and participant informed consent, if relevant	According to local legislation and institutional requirements, ethical review and approval for human participants are not required for this study. Based on national legislation and institutional requirements, written informed consent was not necessary for this study.
5	**Assumptions**	Explicitly state the three core IV assumptions for the main analysis (relevance, independence and exclusion restriction) as well assumptions for any additional or sensitivity analysis	Initially, SNPs demonstrating significant associations with the gut microbiota (P < 1 × 10^-5) were chosen for analysis. SNPs exhibiting linkage disequilibrium (LD) were omitted, with a predetermined threshold of P < 5 × 10^-6. The LD between the identified SNPs and the gut microbiota was required to meet the criteria of r^2 < 0.001 and a distance exceeding 10,000 kb (Myers et al., 2020). A crucial component of MR analysis involves verifying that the impact of an SNP on an individual's exposure aligns with the effect of the SNP on the outcome for the same allele. Following this comparison, palindromic SNPs (defined as those with A/T or G/C alleles) were subsequently excluded from the analysis.
6	**Statistical methods: main analysis**	Describe statistical methods and statistics used	
		a) Describe how quantitative variables were handled in the analyses (i.e., scale, units, model)	To evaluate the potential causal association between the gut microbiota and immune cells in relation to prostate diseases, we conducted independent two-sample MR analyses (steps 1A and 2A in Figure 1).The analytic methods employed were IVW, MR-Egger, weighted median, simple mode, and weighted mode, and Wald ratio tests were conducted for variables featuring only one IVW result (Pierce and Burgess 2013). In cases where discrepancies arose between these methods, the IVW results were considered the primary outcome.

		IVW is a method for calculating a weighted average for random variable analysis. One uses the variance of every random variable as a weight. This approach can help to lower the mean's fluctuation. SNPs are employed to simulate the genetic grouping process.
b)	Describe how genetic variants were handled in the analyses and, if applicable, how their weights were selected	Meanwhile, we use website tools （https://gwas.mrcieu.ac.uk/） to eliminate confounding factors.
c)	Describe the MR estimator (e.g. two-stage least squares, Wald ratio) and related statistics. Detail the included covariates and, in case of two-sample MR, whether the same covariate set was used for adjustment in the two samples	In cases where discrepancies arose between these methods, the IVW results were considered the primary outcome. IVW is a method for calculating a weighted average for random variable analysis. One uses the variance of every random variable as a weight. This approach can help to lower the mean's fluctuation. SNPs are employed to simulate the genetic grouping process. This randomizing strategy allows one to overcome the usual linear character of genetic variation and other potential factors, producing more exact causal inference. The MR–Egger test was utilized to identify outliers and assess horizontal pleiotropy. By considering the similarity in causality, a weighted model technique may categorize SNPs into distinct subsets and utilize the subset with the greatest number of SNPs to evaluate the causal connection between exposure and result. Thus, funnel plots were employed to assess potential directional pleiotropy. To mitigate the potential inaccuracies caused by SNPs, a leave-one-out study was conducted to evaluate the impact of individual SNPs on the MR estimates. Meanwhile, we use website tools （https://gwas.mrcieu.ac.uk/） to eliminate confounding factors. All Mendelian randomization analyses were performed via the ‘Two Sample MR’ (version 0.5.6) package in R version 4.3.1, setting statistical significance at P < 0.05.
d)	Explain how missing data were addressed	In cases where discrepancies arose between these methods, the IVW results were considered the primary outcome. IVW is a method for calculating a weighted average for random variable analysis. One uses the variance of every random variable as a weight. This approach can help to lower the mean's fluctuation. SNPs are employed to simulate the genetic grouping process. This randomizing strategy

		allows one to overcome the usual linear character of genetic variation and other potential factors, producing more exact causal inference. The MR–Egger test was utilized to identify outliers and assess horizontal pleiotropy. By considering the similarity in causality, a weighted model technique may categorize SNPs into distinct subsets and utilize the subset with the greatest number of SNPs to evaluate the causal connection between exposure and result. Thus, funnel plots were employed to assess potential directional pleiotropy. To mitigate the potential inaccuracies caused by SNPs, a leave-one-out study was conducted to evaluate the impact of individual SNPs on the MR estimates. Meanwhile, we use website tools （https://gwas.mrcieu.ac.uk/）to eliminate confounding factors. All Mendelian randomization analyses were performed via the ‘Two Sample MR’ (version 0.5.6) package in R version 4.3.1, setting statistical significance at $P < 0.05$.
	e) If applicable, indicate how multiple testing was addressed	In cases where discrepancies arose between these methods, the IVW results were considered the primary outcome. IVW is a method for calculating a weighted average for random variable analysis. One uses the variance of every random variable as a weight. This approach can help to lower the mean's fluctuation. SNPs are employed to simulate the genetic grouping process. This randomizing strategy allows one to overcome the usual linear character of genetic variation and other potential factors, producing more exact causal inference. The MR–Egger test was utilized to identify outliers and assess horizontal pleiotropy. By considering the similarity in causality, a weighted model technique may categorize SNPs into distinct subsets and utilize the subset with the greatest number of SNPs to evaluate the causal connection between exposure and result. Thus, funnel plots were employed to assess potential directional pleiotropy. To mitigate the potential inaccuracies caused by SNPs, a leave-one-out study was conducted to evaluate the impact of individual SNPs on the MR estimates. Meanwhile, we use website tools （https://gwas.mrcieu.ac.uk/）to eliminate confounding factors. All Mendelian randomization analyses were performed via the ‘Two Sample MR’ (version 0.5.6) package in R version 4.3.1, setting

			statistical significance at P < 0.05.
7	**Assessment of assumptions**	Describe any methods or prior knowledge used to assess the assumptions or justify their validity	This study encompasses three primary components, as depicted in Figure 1: the investigation of the causal effects of 196 gut microbial taxa on three categories of prostate diseases (Step 1A); the analysis of the causal effects of 731 immune cells on three types of prostate diseases (Step 2A); and the implementation of mediation analysis to examine the role of the gut microbiota in mediating the pathway from immune cells to prostate diseases (Step 3). SNPs were defined as instrumental variables (IVs) (Bowden and Holmes 2019).
8	**Sensitivity analyses and additional analyses**	Describe any sensitivity analyses or additional analyses performed (e.g. comparison of effect estimates from different approaches, independent replication, bias analytic techniques, validation of instruments, simulations)	Cochran's Q test was utilized to evaluate heterogeneity for each SNP(Bowden and Holmes 2019), and scatter plots were generated to illustrate the MR results of SNP-exposure and SNP-outcome associations. We employed the stepwise elimination method to assess the individual impact of each SNP pair on the outcomes, wherein we systematically excluded one SNP at a time and applied the IVW method to the remaining SNPs to evaluate the influence of the specific variants on the estimated values.
9	**Software and pre-registration**		
	a)	Name statistical software and package(s), including version and settings used	All Mendelian randomization analyses were performed via the 'Two Sample MR' (version 0.5.6) package in R version 4.3.1, setting statistical significance at P < 0.05.
	b)	State whether the study protocol and details were pre-registered (as well as when and where)	no
	**RESULTS**		
10	**Descriptive data**		
	a)	Report the numbers of individuals at each stage of included studies and reasons for exclusion. Consider use of a flow diagram	After undergoing a rigorous quality control process, the F-statistic values for gut microbiota all exceeded the threshold of >10, suggesting a reduced susceptibility to weak instrument bias. In total, a total of 412 SNPs were identified as being associated with 28 gut microbiota taxa at various taxonomic levels, with a significance level of

		P < 1 × 10–5 (Table S1). Additionally, a total of 1650 SNPs were found to be associated with 75 immune cell types at a significance level of P < 5 × 10–6 (Table S2).
b)	Report summary statistics for phenotypic exposure(s), outcome(s), and other relevant variables (e.g. means, SDs, proportions)	Prostate cancer is associated with ten distinct types of gut microbiota from three classes, two families, two genera, two orders, and one phylum. Detailed information regarding 124 SNPs for these 10 gut microbiota types can be found in Table S3. The MR analysis (Figure 2) indicated a correlation between genetic predictions of five gut microbiota (class Verrucomicrobiae, family Verrucomicrobiaceae, order Verrucomicrobiales, genus Akkermansia, and genus Butyrivibrio) and an elevated risk of PCa. The prevalences of the class Verrucomicrobiae (odds ratio [OR] = 1.2078, 95% confidence interval [CI] = 1.0410-1.4012, P = 0.0127), family Verrucomicrobiaceae (OR = 1.2078, 95% CI = 1.0410-1.4012, P = 0.0127), order Verrucomicrobiales (OR = 1.2078, 95% CI = 1.0410-1.4012, P = 0.0127), genus Akkermansia (OR = 1.2076, 95% CI = 1.0409-1.4009, P = 0.0128), and genus Butyrivibrio (OR = 1.1068, 95% CI = 1.029-1.1904, P = 0.0063) were found to be significantly elevated in cases of PCa. The identification of genetic markers within five distinct intestinal microbiota groups (class Erysipelotrichia, class Mollicutes, family Erysipelotrichaceae, order Erysipelotrichales, and phylum Tenericutes) has been linked to a decreased risk of PCa. The results indicated that the presence of class Erysipelotrichia (OR = 0.7762, 95% CI = 0.6418 ~ 0.9387, P = 0.0090), class Mollicutes (OR = 0.7621, 95% CI = 0.6324 ~ 0.9182, P = 0.0042), family Erysipelotrichaceae (OR = 0.7762, 95% CI = 0.6418 ~ 0.9387, P = 0.0090), order Erysipelotrichales (OR = 0.7762, 95% CI = 0.6418 ~ 0.9387, P = 0.0090), and phylum Tenericutes (OR = 0.7621, 95% CI = 0.6324 ~ 0.9182, P = 0.0042) was associated with a significantly reduced risk of PCa. Importantly, our findings demonstrate heterogeneity, pleiotropy, and sensitivity, including in MR–Egger and weighted median (WM) analyses, which largely corroborated the primary results by consistently indicating the same trends (Figure S1-3). Conversely, reverse MR analysis did not yield any statistically significant

				findings, as evidenced by the results presented in Table S4.
		c)	If the data sources include meta-analyses of previous studies, provide the assessments of heterogeneity across these studies	Importantly, our findings demonstrate heterogeneity, pleiotropy, and sensitivity, including in MR–Egger and weighted median (WM) analyses, which largely corroborated the primary results by consistently indicating the same trends (Figure S1-3).
		d)	For two-sample MR:<br>i. Provide justification of the similarity of the genetic variant-exposure associations between the exposure and outcome samples<br>ii. Provide information on the number of individuals who overlap between the exposure and outcome studies	Additional noteworthy associations were observed with exposures such as dendritic cells, regulatory T (Treg) cells, and myeloid cells. These findings underscore the intricate associations between prostate cancer and diverse cellular markers. However, our examination of pleiotropy and heterogeneity did not yield significant findings. Similarly, in the reverse MR analysis, no significant results were identified (Table S6).
11	**Main results**			
		a)	Report the associations between genetic variant and exposure, and between genetic variant and outcome, preferably on an interpretable scale	MR analysis revealed that 28 gut microbiome taxa and 75 immune cell types were significantly associated with prostate diseases. Furthermore, reverse MR analysis did not support a causal relationship between prostate diseases and the intestinal microbiota or immune cells. Finally, the results of the mediation analysis indicated that Secreting Treg % CD4 Treg, Activated & resting Treg % CD4 Treg, and Mo MDSC AC inhibited the role of the class Mollicutes in reducing the risk of PCa. In prostatitis, CD8+ T cells on EM CD8br hinder the increased risk associated with the genus Eubacterium nodatum group. Interestingly, in BPH, CD28- CD25++CD8br AC and CD16-CD56 on HLA DR+ NK promoted the role of the genus Dorea in reducing the risk of BPH.
		b)	Report MR estimates of the relationship between exposure and outcome, and the measures of uncertainty from the MR analysis, on an interpretable scale, such as odds ratio or relative risk per SD difference	These findings underscore the intricate associations between prostate cancer and diverse cellular markers. However, our examination of pleiotropy and heterogeneity did not yield significant findings. Similarly, in the reverse MR analysis, no significant results were identified.
		c)	If relevant, consider translating estimates of relative risk into absolute risk for a meaningful time period	no

		d)	Consider plots to visualize results (e.g. forest plot, scatterplot of associations between genetic variants and outcome versus between genetic variants and exposure)	yes
12	**Assessment of assumptions**			
		a)	Report the assessment of the validity of the assumptions	In this study, both the intestinal microbiota and immune cells were found to have causal relationships with prostate diseases. The gut microbiota appears to mediate the pathway between immune cells and prostate diseases. Based on the results from the analysis of immune cells on outcomes, we screened gut microbiota immune cells as mediators. We initially selected one intestinal microbial condition with the smallest P value for mediation, but as some intestinal microbial conditions with the smallest P values failed to identify specific immune cell mediators, we continued screening with the second smallest P value. Our results indicated that the class Mollicutes inhibited the role of Secreting Treg % CD4+ Treg, Activated & resting Treg % CD4 Treg, and Mo MDSC AC in reducing the risk of PCa (Table 1).
		b)	Report any additional statistics (e.g., assessments of heterogeneity across genetic variants, such as I^2, Q statistic or E-value)	In this study, both the intestinal microbiota and immune cells were found to have causal relationships with prostate diseases. The gut microbiota appears to mediate the pathway between immune cells and prostate diseases. Based on the results from the analysis of immune cells on outcomes, we screened gut microbiota immune cells as mediators. We initially selected one intestinal microbial condition with the smallest P value for mediation, but as some intestinal microbial conditions with the smallest P values failed to identify specific immune cell mediators, we continued screening with the second smallest P value. Our results indicated that the class Mollicutes inhibited the role of Secreting Treg % CD4+ Treg, Activated & resting Treg % CD4 Treg, and Mo MDSC AC in reducing the risk of PCa (Table 1).
13	**Sensitivity analyses and additional analyses**			

		a)	Report any sensitivity analyses to assess the robustness of the main results to violations of the assumptions	Importantly, our findings demonstrate heterogeneity, pleiotropy, and sensitivity, including in MR–Egger and weighted median (WM) analyses, which largely corroborated the primary results by consistently indicating the same trends (Figure S1-3).
		b)	Report results from other sensitivity analyses or additional analyses	Conversely, reverse MR analysis did not yield any statistically significant findings, as evidenced by the results presented in Table S4.
		c)	Report any assessment of direction of causal relationship (e.g., bidirectional MR)	Report any assessment of direction of causal relationship (e.g., bidirectional MR)
		d)	When relevant, report and compare with estimates from non-MR analyses	Not relevant
		e)	Consider additional plots to visualize results (e.g., leave-one-out analyses)	Figure S3,6,9
	**DISCUSSION**			
14	**Key results**		Summarize key results with reference to study objectives	This study highlights the complex relationships among the gut microbiota, immune cells and prostate diseases. The involvement of the gut microbiota in regulating immune cells to impact prostate diseases could provide novel methods and concepts for its therapy and management.
15	**Limitations**		Discuss limitations of the study, taking into account the validity of the IV assumptions, other sources of potential bias, and imprecision. Discuss both direction and magnitude of any potential bias and any efforts to address them	This study has several limitations. The investigation focused exclusively on the impact of gut microbiota on prostate diseases in the presence of immune cell mediation, potentially overlooking biases arising from other variables. Currently, we merely depend on the GWAS database and don't incorporate additional population data in order to enhance the general applicability of our findings. Furthermore, individual-level variations were not considered for a more in-depth examination of the relationships among gut microbiota, immune cells, and prostate diseases. Finally, because MR analysis depends on unverifiable assumptions, more experimental and clinical evidence is needed to determine how gut microbiota affect prostate disorders through immune cells.
16	**Interpretation**			
		a)	Meaning: Give a cautious overall interpretation of results in the context of their	The objective of this study was to investigate the impact of gut microbiota on prostate diseases,

	limitations and in comparison with other studies	aiming to elucidate its potential therapeutic benefits or detrimental effects. Here, for the first time, we utilized gut microbiota as mediators between the immune cells and prostate diseases. The gut microbiota is a complex and ever-changing system that is impacted by several variables. Simultaneously, as stated in the literature, the disruption of the gut microbiota's ecological equilibrium arises from the imbalance between the gut microbiome and the intestinal epithelium. Intestinal epithelial cells have a crucial function in preserving the mutually beneficial interaction between the gut microbiota and the host. They do this by creating a protective layer of mucus, releasing different immunological substances, and conveying bacterial antigens (Shi et al., 2017, Okumura and Takeda 2017, Liu et al., 2020). On the other hand, our research findings indicate that alterations in the gut microbiota can impact the maturation of immune cells, specifically B cells, Treg cells, Myeloid cells, and Classical dendritic cells, thereby affecting prostate diseases (Figure 8). These findings align with the studies conducted by Ji-Eun Kim, Leonie Brockmann, Baichao Yu, and other scholars (Kim et al., 2022, Brockmann et al., 2023, Yu et al., 2021). Using mediation analysis, we revealed that various immune cell subsets, including Secreting Treg % CD4 Treg, Activated & resting Treg % CD4 Treg, Mo MDSC AC, CD8 on EM CD8br, CD28- CD25++ CD8br AC, and CD16-CD56 on HLA DR+ NK, exert both protective and detrimental effects on prostate diseases by modulating the abundance of specific bacterial taxa, such as class Mollicutes, genus Eubacterium nodatum group and genus Dorea.
b)	Mechanism: Discuss underlying biological mechanisms that could drive a potential causal relationship between the investigated exposure and the outcome, and whether the gene-environment equivalence assumption is reasonable. Use causal language carefully, clarifying that IV estimates may provide causal effects only under certain assumptions	Meaning: Give a cautious overall interpretation of results in the context of their limitations and in comparison with other studies<br>Mechanism: Discuss underlying biological mechanisms that could drive a potential causal relationship between the investigated exposure and the outcome, and whether the gene-environment equivalence assumption is reasonable. Use causal language carefully, clarifying that IV estimates may provide causal effects only under certain assumptions

		c) Clinical relevance: Discuss whether the results have clinical or public policy relevance, and to what extent they inform effect sizes of possible interventions	The connection between gut microbiota and prostatitis is complex, as evidenced by prior research demonstrating a significant reduction in gut microbiota diversity in individuals with chronic prostatitis (Shoskes et al., 2016). The present study findings substantiated the relationship between four gut microbiota taxa (family Prevotellaceae, genus Eubacterium nodatum group, genus Ruminococcaceae NK4A214 group, and order Rhodospirillales) and susceptibility to prostatitis. Previous studies have established a correlation between dysregulated gut microbiota composition and depressive-like behaviour in mice afflicted with experimental autoimmune prostatitis (EAP). The findings of subsequent investigations revealed that the gut microbiota can modulate short-chain fatty acid production, thereby impacting Th17/Treg cell differentiation in mice with EAP (Du et al., 2022). This finding is further supported by findings from an EAP model in which hyperactivation of Th1 and Th17 cells was observed (Zhan et al., 2020, Chen et al., 2022, Murphy et al., 2015). Alternatively, Th1 cells can exert their effects by migrating to the prostate site through the expression of CXCR3 (Breser et al., 2013, Yue et al., 2023). Our study findings revealed a positive correlation between myeloid cells and susceptibility to prostatitis, while CD4 Treg %CD4, Activated & resting Treg % CD4+, and CD25hi %T cell in Treg cells were identified as suppressors of this risk. These findings suggest that Treg cells play a protective role in prostatitis patients. Additionally, our results indicated that in prostatitis, CD8 on EM CD8br may impede the increased risk posed by the genus Eubacterium nodatum group.
17	**Generalizability**	Discuss the generalizability of the study results (a) to other populations, (b) across other exposure periods/timings, and (c) across other levels of exposure	Recent research has shown that changes in the gut microbiota significantly impact the pathogenesis, progression, diagnosis, and management of BPH. Specifically, the presence of Lactobacillus, Flavonifractor, and Acetatifactor in the BPH model has been linked to key indicators of this condition (An et al., 2023). Xia established a causal relationship between gut microbiota and BPH via MR analysis. These findings revealed significant associations between BPH and the presence of Eisenbergiella, Ruminococcaceae (UCG009), and

			Escherichia shigella (Xia et al., 2023). The findings of this study revealed that the Ruminococcaceae NK4A214 group, Sellimonas genus, and Verrucomicrobia phylum did not exhibit a protective effect on BPH. Further investigation and confirmation are required to elucidate the mechanisms through which gut microbiota influences the pathogenesis of BPH. Chronic inflammation in BPH can perpetually stimulate the prostate gland, thereby impacting the onset and progression of BPH (Nickel et al., 2017). M2 macrophages are the predominant inflammatory cells that infiltrate and proliferate within the prostate gland. The secretion of cytokines and growth factors plays a crucial role in driving the pathogenesis of BPH(Gandaglia et al., 2017). CD8+ T cells are commonly found in the periglandular region surrounding the epithelial tubes, whereas lymphoid aggregates consisting of B lymphocytes and follicular T lymphocytes are located within the fibromuscular stroma(De Nunzio et al., 2011). BPH cells can augment the inflammatory response by recruiting additional inflammatory cells through diverse mechanisms (Cao et al., 2022). Our study findings suggested that B cells play a protective role in the pathogenesis of BPH. Conversely, there is an intricate interplay between Treg cells, myeloid cells, and TBNK cells and BPH. Our findings demonstrate that in BPH, certain subsets of immune cells, such as CD28- CD25++ CD8br antigen-presenting cells and CD16-CD56 natural killer cells expressing HLA DR+, contribute to a reduced risk of colonization by the genus Dorea. However, further investigation is required to identify how gut microbiota modulate immune cell responses and subsequently impact BPH.
	**OTHER INFORMATION**		
18	**Funding**	Describe sources of funding and the role of funders in the present study and, if applicable, sources of funding for the databases and original study or studies on which the present study is based	This work was supported by the National Natural Science Fund of China (82170787, 82100815 and 82272886) , Anhui Natural Science Foundation (2108085QH315), Natural Science Foundation of Anhui Education Department (2022AH030118)， Research Fund of Anhui Institute of Translational Medicine (2022zhyx-C37).

19	**Data and data sharing**	Provide the data used to perform all analyses or report where and how the data can be accessed, and reference these sources in the article. Provide the statistical code needed to reproduce the results in the article, or report whether the code is publicly accessible and if so, where	Methods
20	**Conflicts of Interest**	All authors should declare all potential conflicts of interest	The authors declare that there are no conflicts of interest.

This checklist is copyrighted by the Equator Network under the Creative Commons Attribution 3.0 Unported (CC BY 3.0) license.

1. Skrivankova VW, Richmond RC, Woolf BAR, Yarmolinsky J, Davies NM, Swanson SA, et al. Strengthening the Reporting of Observational Studies in Epidemiology using Mendelian Randomization (STROBE-MR) Statement. JAMA. 2021;under review.

2. Skrivankova VW, Richmond RC, Woolf BAR, Davies NM, Swanson SA, VanderWeele TJ, et al. Strengthening the Reporting of Observational Studies in Epidemiology using Mendelian Randomisation (STROBE-MR): Explanation and Elaboration. BMJ. 2021;375:n2233.
